# Exploring the impact of a brief positive experience on dogs’ performance and stress resilience during a learning task

**DOI:** 10.1371/journal.pone.0326368

**Published:** 2025-06-23

**Authors:** Julia Miller, Camila Cavalli, Amin Azadian, Alexandra Protopopova

**Affiliations:** 1 Animal Welfare Program, Faculty of Land and Food Systems, The University of British Columbia, Vancouver, Canada; 2 Department of Immunology, Pathophysiology and Veterinary Preventive Medicine, University of Environmental and Life Sciences, Wroclaw, Poland; Universidade do Porto Instituto de Biologia Molecular e Celular, PORTUGAL

## Abstract

Learning and stress resilience can be influenced by recent experiences. Research has traditionally focused on the effects of negative situations and stressors on subsequent learning and stress resilience, while knowledge is limited regarding the effects of positive experiences. We aimed to examine the impact of a pre-session brief positive experience on dogs’ learning and stress resilience. Pet dogs were quasi-randomly assigned to the experimental (n = 20) or control (n = 20) group, counterbalanced for age, sex, and breed clade. Experimental dogs received a session intended to provide a positive experience, which included a 15 min walk on a long leash, human interaction, exploration, playing, and olfactory-based foraging. Control dogs were kept on leash in an office without being allowed to explore nor interact with their owner or the experimenters for 15 min. After 60 s of habituation to the testing room, all dogs were taught to nose-touch the experimenter’s hand. After the Learning phase, there was a 2 min Disruption phase, in which a remote-controlled car moved inside of a tub at a distance. Measures included the number of hand touches in each phase, the latency to return to the task, and general stress and affiliative behaviours. No differences were observed in the Learning phase. Surprisingly, experimental dogs exhibited higher stress levels than control dogs during the Disruption phase. These dogs also spent a significantly higher proportion of time in proximity to their owners, which could be interpreted as reassurance-seeking behaviour. Contrary to our predictions, exposure to a brief positive experience did not impact learning and, surprisingly, seemed to have made dogs more susceptible to stress during the Disruption phase. Several possible explanations are discussed, including the possibility of an unintentional induction of a negative emotional state by the termination of the positive experience, as well as differences in arousal, or habituation to the indoor environment.

## Introduction

The domestication history of dogs has resulted in their adaptation to the human social environment, trainability, and the development of understanding of communicative cues given by humans [1–4]. Despite this adaptation, living in a human-dominated environment can be associated with many challenges, including frequent encounters with unfamiliar objects, sounds, people, and other animals.

A key goal in improving the welfare of companion dogs is to understand how to create behavioural interventions in order to support dogs’ resilience to these stressors. The definition of ‘resilience’ is continuously evolving and is typically thought of in terms of human functioning [5]. However, in a non-human animal context, resilience, while still having many definitions, may be defined as being able to cope with an environmental stressor [reviewed in 6,7]. Therefore, in a behavioural context, resilience may be assessed by the ability of the animal to “bounce back” following an administration of a stressor [7].

A dog’s response to environmental stressors depends on individual traits shaped by multiple factors, such as genetics, early socialisation, and prior experience [e.g., 8–13]. Moreover, as noted by dog training professionals, dogs may also experience the phenomenon of “trigger stacking”, in which their resilience to a relatively mild stressor is reduced due to an accumulation of prior stressors [14,15].

A variety of tests have been implemented to assess canine stress, including exposure to various ambiguous or novel stimuli (e.g., a vacuum cleaner, a startling sound, or a remote-controlled toy car [16–20]), and social situations (e.g., being left alone, being ignored by the owner, being alone with a neutral or friendly stranger, or even encountering a stranger exhibiting threatening behaviour [19,21–25]). The evaluation of dogs’ stress during these tasks includes behavioural assessments and measurement of physiological parameters such as cortisol or heart rate [16,19,20,25].

Due to the unique human-dog relationship and the variety of roles dogs play in our society (e.g., pet, assistance, therapy, guarding), another field of extensive research focuses on learning capacity and the factors influencing the outcomes of dogs’ training. These factors include genetics, training history, early experiences, age, and source of acquisition [26–30]. Nevertheless, learning is also likely impaired by stress [for a review see: 31,32]. For instance, research has shown that shelter dogs tend to perform worse than pet dogs in a variety of learning tasks, which has been attributed to their reduced experience interacting with people, along with the substantial stress they endure as a result of these living conditions [33–36]. Other animal studies have experimentally demonstrated the influence of aversive stimuli on cognitive performance. For example, social isolation and unfamiliar environments negatively affected pig performance in a spatial memory test [37]. Similarly, moving to a new environment temporarily reduced dwarf goats’ performance in a visual shape discrimination task [38]. In sheep, pre-treatment with threatening stimuli and presenting white noise during the task negatively affected their performance in a spatial maze task [39]. Interestingly, in a study evaluating the performance of both owners and their dogs in a spatial working memory task, dogs who were stressed by being separated from their owners showed better performance [40]. Given the relatively scarce literature on this topic, more research is needed to increase our understanding of the effects of stress on different types of learning tasks in companion animals.

The above-mentioned studies are part of a larger body of research focused on how animals respond to situations expected to induce specific emotional states, usually related to fear and anxiety [41].

Given that a negative experience may hinder subsequent learning, it becomes relevant to examine if positive experiences, instead, may enhance learning. However, there has been less focus on the potential influence of positive experiences on animal learning [but see 42]. To date, evidence for the impact of a positive affective state on different aspects of learning comes mainly from human studies. However, the results of these studies are mixed. While some show a beneficial impact of a positive affective state on different aspects of cognition, such as creative problem-solving or knowledge transfer [e.g., 43,44], others show opposite or mixed effects [e.g., 45,46]. Ways of inducing affective states in human participants include a pre-task treatment phase, such as the presentation of comedy videos versus neutral videos or videos with disturbing content [e.g., 43,45], performing a self-referencing mood-induction procedure [47], or a self-induced mood manipulation [48].

Whereas administering pain, restraint, or isolation is easy to conceptualize as “negative” for an animal and these experiences are often included in studies on animal welfare, procedures that might induce a positive state in an animal are studied much less frequently. Providing enrichment, such as additional sensory stimulation and positive human interaction has been demonstrated to result in general benefits to many animals [for a review see 49,50] and olfactory stimulation was shown to positively impact the behaviour of kenneled dogs as was shown by increased exploration and reduced stress-related behaviours [51]. A test widely used for evaluating emotional states (more precisely, optimism and pessimism) is the judgment bias test [for a review, see 52]. Briefly, this test focuses on the animal’s behaviour towards an ambiguous stimulus, for instance, a bowl placed halfway between two locations previously established as positive (always contains food) or negative (always presented empty). The dog’s latency to approach the ambiguous location is considered an indicator of their positive (optimistic) or negative (pessimistic) affective state, as it would be expected for them to approach faster if they are expecting food in the ambiguous location. Duranton and Horowitz [53] found that engaging dogs in olfactory-based activity (i.e., “nosework”) for two weeks resulted in a more optimistic approach compared to the pre-session baseline. Olfactory enrichment with essential oils also generated a more optimistic response in shelter dogs [54]. Positive human interaction increased optimistic approach in fearful dogs housed in an animal shelter (however, the same procedure did not result in changes in optimism in non-fearful dogs) [55]. The provision of a complex toy resulted in a slightly more optimistic approach in pet dogs, albeit the effect might not have been robust [56]. Nevertheless, the data on the induction of positive affective states and their subsequent impact on dogs’ behaviour and learning remains limited.

This study aimed to evaluate the impact of a brief positive experience (including exploration, playing with a toy, and olfactory-based foraging) on dogs’ learning and subsequent stress resilience in the presence of a potentially stressful stimulus. We hypothesised that a pre-session positive experience, compared to a neutral experience, would result in better performance in the learning task and increased stress resilience, evidenced by a shorter latency to return to the learning task after the disruption began.

## Materials and methods

### Ethical statement

**Fig 1:**
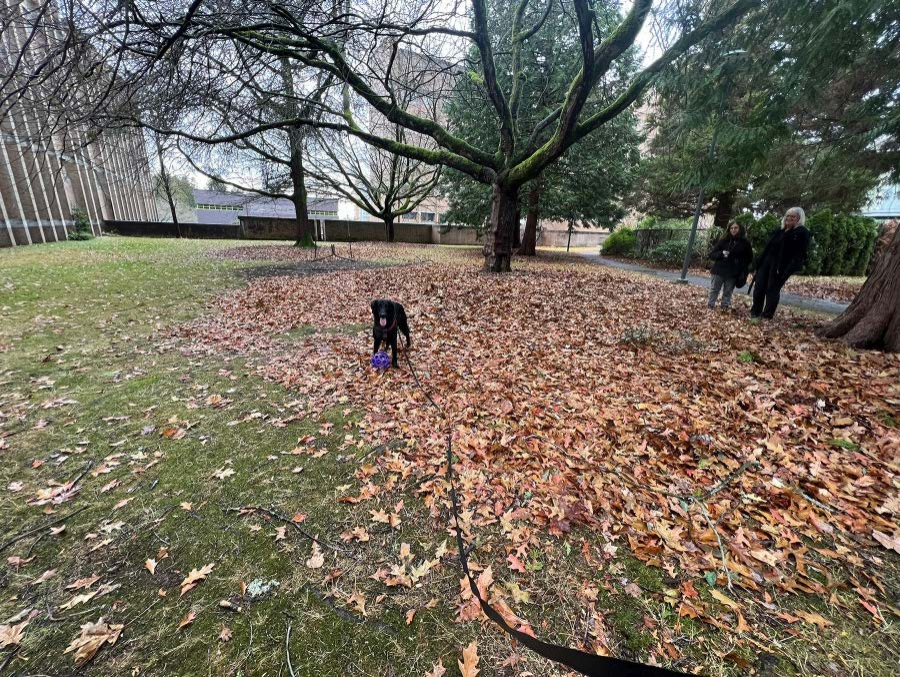
Image of the setup in the experimental condition. The structured walk consisted of 5 min of free exploration, 5 min of playing with a toy, and 5 min of olfactory-based foraging.

**Fig 2:**
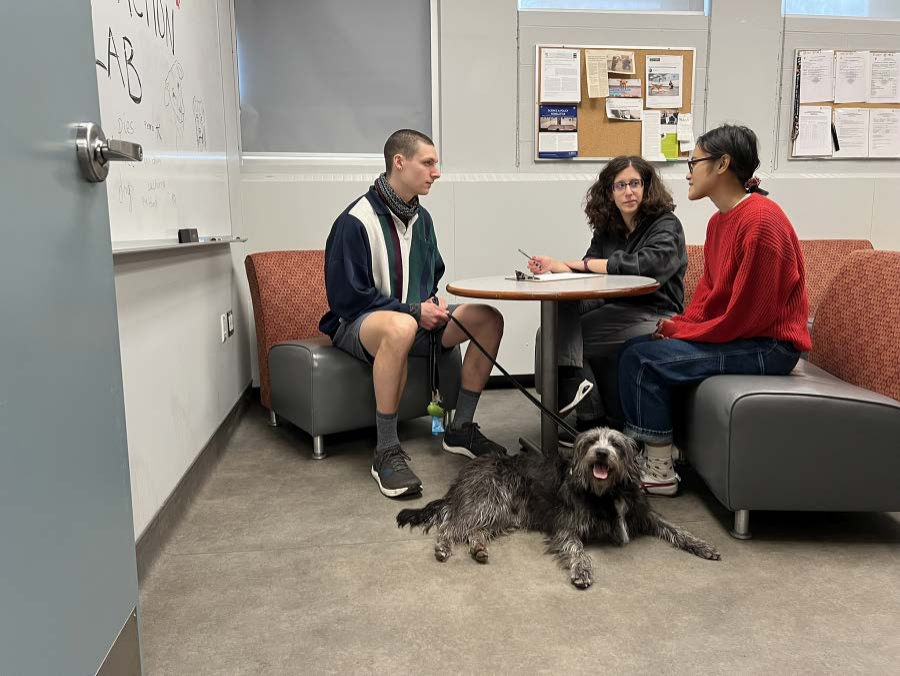
Image of the setup in the control condition. The dogs stayed with the owner and the experimenters in an office space.

All procedures were approved by the University of British Columbia Animal Care Committee (A22-0170). Owners consented to the participation of their dogs in this study. No human-related data were obtained nor analysed during the study. The individuals seen in Figs 1 and 2 have given written informed consent (as outlined in PLOS consent form) to publish these pictures.

### Subjects

Fifty-two dogs were recruited to the study. The inclusion criteria for the study included being comfortable in unfamiliar environments and around strangers, as reported by the owner, not being already familiar with the hand touch command, having high food motivation as reported by the owner, having current core vaccinations, and being between six months and ten years of age. Twelve dogs (eight neutered males and four neutered females; mean age = 4.67 ± 2.67 years) were excluded from the study because of early termination of the experiment due to fearfulness (n = 7, four from the experimental group) or not meeting the learning criteria of the task (n = 5, three from the experimental group). Forty pet dogs (mean age = 4.42 ± 3.05 years) were quasi-randomly assigned to the experimental (n = 20) or control (n = 20) groups, counterbalanced for age, sex, and breed clade (following Parker et al. [57]). The experimental group (mean age = 4.12 ± 2.18 years) included six neutered females, one intact female, 12 neutered males, and one intact male. The control group (mean age = 4.75 ± 2.59) consisted of eight neutered females, 11 neutered males, and one intact male. See Table 1 for detailed information on the dogs recruited for the study.

**Table 1:** Identification numbers (ID No), breed, age, sex, and neuter status of dogs recruited into the study.

Control group				Experimental group			
ID No	Breed	Age (years)	Sex	ID No	Breed	Age (years)	Sex
C2	Labrador Retriever	5	spayed female	E1	Mixed breed	4	neutered male
C3	Mixed Breed	2	neutered male	E3	Standard Poodle	4	neutered male
C4	Labrador Retriever	8	neutered male	E6	Golden Retriever	2	spayed female
C5	Bernese Mountain Dog	0.5	intact male	E7	Mixed breed	8.5	neutered male
C6	Vizsla	7.5	neutered male	E8	Mixed breed	5	neutered male
C7	Portuguese Water Dog	4	spayed female	E10	Mixed Breed	0.5	neutered male
C8	Toy Poodle	2	neutered male	E11	Toy Poodle	4	neutered male
C9	Welsh Corgi Pembroke	6	spayed female	E12	American Bully	8	neutered male
C11	Beagle	5	spayed female	E13	Mixed breed	7	neutered male
C12	Mixed Breed	3	neutered male	E15	Dachshund	7	spayed female
C13	Mixed Breed	2	neutered male	E16	West Highland WhiteTerrier	3	neutered male
C14	Golden Retriever	4	neutered male	E17	Mixed breed	6	spayed female
C17	Biewer Terrier	2	neutered male	E18	Mixed breed	2	spayed female
C18	Mixed Breed	1.5	spayed female	E19	Flat Coated Retriever	6	spayed female
C19	Mixed Breed	1.5	spayed female	E22	Golden Retriever	5	neutered male
C20	Mixed Breed	3	spayed female	E23	Labrador Retriever	0.75	intact female
C22	Mixed breed	4	neutered male	E24	Welsh Corgi Pembroke	1	spayed female
C23	Welsh Corgi Pembroke	9	neutered male	E25	Border Collie	2	neutered male
C24	Duck Tolling Retriever	10	neutered male	E26	Bernese Mountain Dog	3	neutered male
C25	Bernese Mountain Dog	7	spayed female	E27	Wirehaired Pointing Griffon	1	intact male

Owners of the dogs who reached the learning criteria completed the Training and Obedience, Fear and Anxiety, and Attachment and Attention-seeking subscales of the Canine Behavioral Assessment and Research Questionnaire (C-BARQ, Hsu & Serpell [58]).

### Experimental conditions

For the experimental condition, dogs received a 15-min structured walk in a quiet outdoor area with their owner and two experimenters. See Fig 1.

The walk consisted of 5 min of free exploration, 5 min of playing with a toy (tug toy or ball, depending on the dog’s engagement with the toy), and 5 min of olfactory-based foraging (sniffing for treats [Zuke’s Mini Naturals®] scattered in the grass). The handler repeatedly tried to engage the dog in play, while the experimenter, who would later conduct the learning task, did not encourage interaction (i.e., not talking to the dog nor petting them if approached). If the dogs were not interested in any of the available toys, they continued with free exploration instead. The activities were chosen, based on previous research that found that human social interaction, access to toys, and olfactory search induce ‘optimism’ in dogs [53,54]. To encourage olfactory search, and given that dogs benefit from food-stuffed toys [59] we added treats as one of the elements of the positive experience session. The sequence of the tasks remained consistent across dogs. Exploration occurred first to allow for habituation to the experimenter and olfactory foraging was last to minimise potential frustration from removing access to food.

Dogs were fitted with a 4 m long leash held by one of the experimenters (i.e., handler) to ensure that the leash remained loose to reduce any potential sensation of physical restriction. All authors have extensive experience working professionally with dogs and utilised their skills to reduce any potential discomfort and increase positive affect (through observing dog body language and adjusting movements accordingly).

For the control condition, dogs spent 15 min with their owner and the two experimenters in an office space with three chairs and a table. During this time, the experimenters held a general conversation with the owners, and the owners were given the C-BARQ questionnaire to complete. If the owners did not finish completing the questionnaire during this part of the study, they continued after the experiment (similarly, owners of the experimental group dogs completed the C-BARQ questionnaire after the experiment). The dogs were kept on leash without being allowed to explore the room, and all attempts to interact with the people were ignored. Dogs in this condition did not receive any treats. See Fig 2.

The experimenters were JM or CC for all dogs. The handlers were usually CC or AP, except on three occasions when the handler was AA.

### Task setup

**Fig 3:**
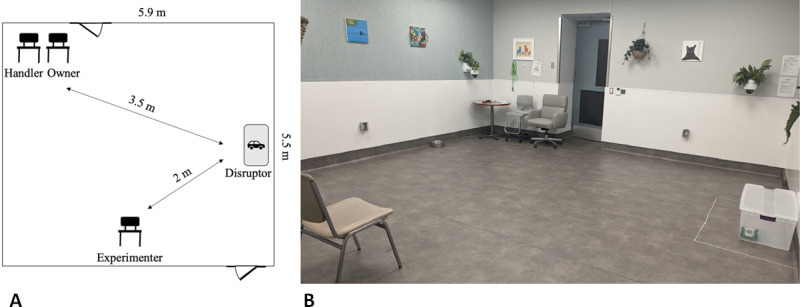
Drawing (A) and image (B) of the setup during the task. The handler and the owner sat in the corner of the room. The plastic tub containing a remotely controlled car was placed on the other side of the room. The experimenter sat in the centre of the room, approx. 3.5 m away from the owner and the handler.

The owner and the handler (or one of the experimenters from the control condition) sat in the corner of the room. The experimenter, responsible for teaching the hand-touch command, sat in the centre of the room (on a chair or the floor, depending on the dog’s size), approx. 3.5 m away from the owner and the handler. A water bowl was located next to the wall opposite the tub, close to the owner. A plastic transparent tub (width 88 cm x depth 48 cm x height 32 cm) containing a remotely controlled car (LiteHawk REBEL®) was placed on the other side of the room, approximately 3.5 m away from the owner and the handler, and approximately 2 m away from the experimenter. A perimeter was marked on the floor at 30 cm from the tub and was later used to evaluate the proximity to the car during the analyses of the videos. See Fig 3.

The experimenter used her left or right hand to teach the hand touch command, depending on the owner’s dominant hand. In the case of right-handed owners, the dogs were trained to touch the experimenter’s left hand; in the case of left-handed owners (dogs’ IDs: C4, E4), the dog was trained to touch the experimenter’s right hand. Additionally, the tub containing the car was placed on the right or left side of the room to ensure each dog would touch the hand further from the tub. The handler counted the time with a stopwatch and gave the sign to switch on the remotely controlled car that would be used as a disruptor (see Procedure) but did not interact with the dog.

All people were wearing dark sunglasses to avoid unintentional cueing caused by human gazing. The situation was filmed using a closed video circuit comprising four video cameras placed in each corner of the room.

The rewards for the task were small pieces of MaplelodgeFarms® chicken hot dog (each hot dog was sliced approximately 0.5 cm thick, and then each slice was cut into quarters to get several small treats). The rewards for six experimental (E1, E3, E6, E11, E16, E24) and seven control dogs (C2, C3, C4, C5, C8, C18, C23) were either Zuke’s Mini Naturals® pieces in salmon flavour or other owner-provided commercial treats due to allergy concerns.

### Procedure

After the pre-treatment, all dogs were brought into the testing room and received a 60 s habituation phase. During this time, dogs were let off-leash and were free to explore the room while the experimenters explained the next phase of the task to the owners.

#### Learning phase.

Immediately afterwards, the experimenter called the dog’s name and fully extended her arm to the side, presenting the palm of her hand at the height of the dog’s head. If the dog touched the hand (with any part of the body), the experimenter marked the behaviour with a verbal “yes” and tossed a treat on the floor in front of her. After the dog gazed back toward the experimenter, a new trial began, and the hand was presented again.

The learning criteria were met if the dog performed six subsequent touches with a latency of less than 3 s from the presentation of the hand to the touch. If the dog did not touch the hand within 10 s, the trial was considered a “no-choice”, and the experimenter withdrew her hand and presented it again. After two consecutive no-choices, the experimenter presented her hand while holding a treat between her fingers to encourage the dog to approach it (i.e., assisted trial). Once the learning criteria were reached, the handler gave a sign (thumbs up) to a helper waiting in the control room to turn on the remotely controlled car in the plastic tub.

#### Disruption phase.

In the Disruption phase, the experimenter continued to present her hand to the dog, while the car was switched on for 2 minutes. The helper was instructed to move the car erratically within the tub but refrain from constantly banging on the walls. The experimenter presented her hand immediately after the car was switched on and kept her arm extended continuously until the dog touched her hand for the first time. If that happened, the behaviour was again marked with a “yes” and rewarded with a treat, and the task continued as described in the Learning phase. In one case (dog’s ID: E16), the car was switched off after 55 s because of excessive stress, as was evidenced by the body language and vocalisations (barking) of the dog.

#### Food motivation control.

After the car was switched off, the experimenter waited 30 s without interacting with the dog. After this time, she tossed ten treats on the floor in front of her to evaluate food motivation at the end of the experiment. All dogs ate all of the treats during this phase. The purpose of this phase was to ensure that the dogs’ engagement (or lack of engagement) in the task was not predominantly impacted by satiety.

### Data analysis

For the Learning phase, the “number of hand touches needed for acquisition”, “number of no-choices”, “number of assisted trials”, and “latency to acquire the behaviour” were analysed. In the case of two dogs from the experimental group (E1, E18), the Learning phase included a short break in which the dog could freely explore the room again, as the dogs initially showed no interest in the interaction with the experimenter. For these two dogs, we have not analysed “latency to acquire the behaviour”. For the Disruption phase, the “latency to return to the task” (first hand touch after switching on the car) and “number of hand touches” were analysed. Additionally, for both phases, videos were coded for affiliative behaviours toward the owner and the handler, body language related to stress, barking, and other behaviours (proximity to the door, sitting, lying down). For the Disruption phase, the videos were additionally coded for behaviours toward the stressor, and affiliative behaviours toward the experimenter. Note that affiliative behaviours toward the experimenter were not coded in the Learning phase because it was impossible to distinguish the dog’s motivation, e.g., for gazing at the experimenter at the time when they did not know the task and were engaged in learning. The coding was conducted using a partial-interval procedure with 5 s time bins. Occurrence or non-occurrence in each 5 s time bin was noted for each behaviour to accommodate a balance between recording event and state behaviours and allowing for the comparison across behaviours [60]. Due to a technical problem, sound was not correctly recorded for 12 dogs. For this reason, instead of coding all vocalisations, we decided to analyse only barking, as this behaviour can also be registered based on the dog’s facial movements. See Table 2 for a full description of the analysed behaviours. The results are expressed as a proportion of the time bins in which each behaviour occurred. Because of the treat types used (soft pieces of chicken hot dogs) the lip-licking behaviour was counted as linked to stress only if it occurred in a time bin without food delivery. Single elements of body language were subsequently presented together as an “average stress score/time bin” (by summing up the number of unique behaviours that were scored as present in a given time bin and thus presenting the average stress ‘score’ per time bin).

**Table 2:** Operational definitions of the behaviours that were observed during the Learning and Disruption phase.

Behaviour	Operational definition
**Affiliative behaviours**	
**Owner/handler proximity**	At least one paw placed within arm’s length from the owner and/or handler
**Experimenter proximity**	At least one paw placed within arm’s length from the experimenter while the dog is not participating in the task
**Gazing at owner/handler**	The eyes of the dog are directed at/in the direction of the owner and/or handler
**Gazing at experimenter**	The eyes of the dog are directed at/in the direction of the experimenter while the dog is not participating in the task
**Physical contact owner**	Any form of body contact with the owner initiated by the dog, including placing paws, leaning, or sniffing
**Physical contact handler**	Any form of body contact with the handler initiated by the dog, including placing paws, leaning, or sniffing
**Physical contact experimenter**	Any form of body contact with the experimenter initiated by the dog, including placing paws, leaning, or sniffing while the dog is not participating in the task
**Behaviours related to the disruptor**	
**Approaching the car**	Distance between the dog and the car is decreasing while the dog is not participating in the task
**Retreating from the car**	Distance between the dog and the car is increasing while the dog is not participating in the task
**Startle**	A sudden, sharp movement of the whole body
**Car proximity**	At least one paw or the head is within or on the tape measuring out 30 cm from the box with the car
**Gazing at the car**	The eyes of the dog are directed at the car
**Other behaviours**	
**Barking**	Opening the mouth with a rapid, rhythmic burst of movement
**Sitting**	The body is supported by two extended front legs and two flexed back legs
**Lying down**	The dog is lying down with limbs either tucked under or placed in front of the body
**Door proximity**	Any part of the dog is at arm’s length or closer away from the door, the body oriented toward the door, including physical contact with the door (scratching, jumping, sniffing)
**Food delivery**	The dog is eating a treat
**Body language linked to stress**	
**Yawning**	Opening the mouth wide for at least 1 s
**Panting**	Breathing rapidly through opened mouth
**Lip licking**	Licking over lips or nose
**Shaking off**	Movements of body and/or head back and forth repeatedly and rapidly
**Paw lift**	Any paw is lifted off the ground for 1 s or more
**Tail down**	The tail is held between or through the hind legs or is forcibly held down (the base of the tail pressed to the body)
**Cowering**	The body is in a lowered, crouched position
**Hiding**	Hiding under a chair or table

One observer coded all videos, while a second observer coded 30% of randomly selected videos. For most behaviours, the inter-observer agreement was high (range: 95.1% – 100%, mean: 97.4% ± 3.2%, min: 95.1% for “gazing at the car”, and max: 100% for “cowering”, “yawning”, “shaking off” and “lying down”). The highest discrepancies were seen in coding “tail down” (58.3% − 100%), therefore this behaviour was double-coded in all of the videos from the Disruption phase with a mean agreement of 96.2% ± 8.1%.

To make sure that no single behaviour was over-interpreted as indicative of stress during the ethogram-based coding, both observers also scored the dogs’ stress levels during the Disruption phase on a holistic 4-point scale (0 – no stress, 1 – low stress, 2 – moderate stress, 3 – high stress) based on their overall body language, barking, and other behaviours (i.e., seeking the proximity of the owner, and behaviour towards the object; see Table 3). The inter-observer agreement for this analysis was near perfect (Cohen’s kappa = .96).

**Table 3:** Definitions used in the overall holistic stress assessment scale.

Score	Definition
**0 (no stress)**	The dog shows no signs of stress, or there is only a brief, mild reaction after turning on the car; the dog goes back to the task or lies down in the room; if looking at the car, the dog shows no signs of stress.
**1 (low stress)**	The dog shows mild signs of stress after turning on the car; the reaction lasts longer than a few seconds or occurs repeatedly during the whole phase; for the majority of the phase, the dog engages in the task or seems to be rather distracted by the car than scared of it.
**2 (moderate stress)**	The dog shows stress signs during the majority of the phase or intense stress signs after the car was turned on; even if engaging in the task for the majority of the phase, the dog is cautious and tense; the dog may repeatedly look at the car showing stress signs and/or look for reassurance in the proximity of the owner.
**3 (high stress)**	The dog shows intense stress signs and/or is continuously looking for reassurance in the proximity of the owner while exhibiting stress signs; the dog touches the experimenter’s hand only a few times or does not engage in the task at all.

The stress level in the Disruption phase could be influenced not only by our experimental condition but also by the Learning phase, which took place directly before the Disruption phase. Therefore, a correlation was calculated to see whether there was a relationship between the “number of touches needed to acquisition” or “latency to acquire the behaviour” (which could contribute, for example, to longer habituation to the room or, on the contrary, frustration by lower success rate during the Learning phase) and the stress measurements of the Disruption phase.

#### Statistical analyses.

**Fig 4:**
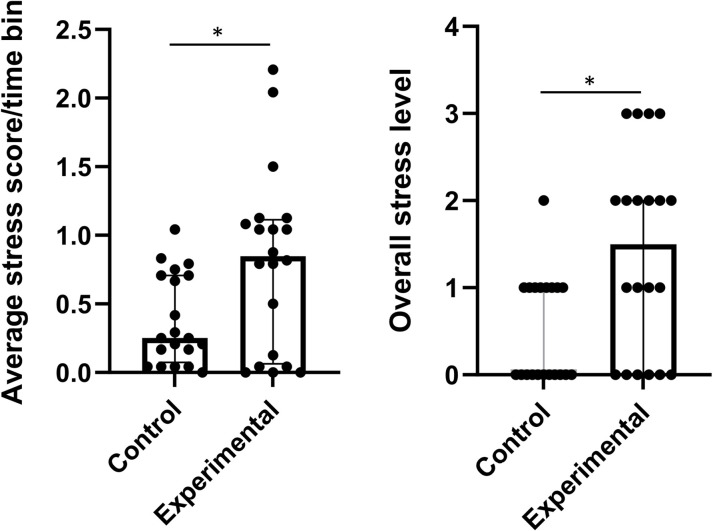
Overall stress level and average stress score/time bin. Median (box) and interquartile range (whiskers) of overall stress level and average stress score/time bin in the control and experimental group. Dots represent individual values. * *p *< 0.05.

Statistical analyses were conducted in Statistica 13 (StatSoft Tulsa, USA). A T-student test was used for normally distributed data (CBAR-Q Fear subscale; CBAR-Q Attachment subscale, “number of hand touches” in the Disruption phase). Non-parametric analyses (Mann–Whitney *U* test to compare the two groups and Wilcoxon signed-rank test to compare the results in the Learning phase vs the Disruption phase within one group) were used for the rest of the data, that were not normally distributed (*p* < .05 in the Shapiro-Wilk test). The differences were considered significant if *p* < .05. Spearman’s Rank correlation coefficient was used to analyse the relationship between the learning-related measurements in the Learning phase and the Disruption phase, as well as to analyse the relationship between the results of stress evaluation based on behavioural coding (“average stress score/time bin”) and “overall stress level”. Cohen’s kappa was used to analyse the inter-observer agreement in the overall stress level assessment. The inter-observer agreement for individual behaviours was calculated by summing all agreements of whether or not a behaviour occurred in that interval, dividing it by the number of intervals, and multiplying by 100. Fig 4 was created with GraphPad Prism 10.1.2. Raw data used for the analyses are presented in S1 Dataset.

## Results

### C-BARQ scores

The results of the C-BARQ questionnaires are presented in Table 4. There were no statistical differences between the groups.

**Table 4:** Results of the C-BARQ subscales.

	Fear & Anxiety		Non-social Fear		Training & Obedience		Attachment	
	Mean ±SD	Student’s t test	Median (IQR)	MWU test	Median (IQR)	MWU test	Mean ±SD	Student’s t test
**Control**	4.03 ±2.38	*t *= 0.74 *df* = 38 *p *= .462	0.67 (1.00)	*U* = 149.50 *p* = .174	2.56 (0.56)	*U* = 165.50 *p* = .355	2.44 ±0.70	*t *= 0.70 *df* = 38 *p *= .290
**Experimental**	3.49 ±2.27		1.00 (1.25)		2.81 (0.63)		2.21 ±0.67	

Student’s t test was used for normally distributed data; Mann Whitney U (MWU) test was used for data that were not normally distributed. No statistical differences were noted between the groups.

### Learning phase

No statistical differences were found between the control and the experimental group in the “number of hand touches needed to acquisition”, “number of no choices”, “number of assisted trials”, nor “latency to acquire the behaviour”. Similarly, no differences were significant in other parameters, including the “average stress score/time bin”, affiliative behaviours, as well as “sitting”, “lying down”, and “barking”. The proportion of time bins where the dogs stayed in the door proximity was significantly higher in the experimental group. See Table 5 for detailed results.

**Table 5:** Stress-related, affiliative and task-related parameters, as well as other behaviours measured during the Learning phase.

		Control	Experimental	Statistics
	Median (IQR)	Median (IQR)	MWU test
**Stress-related parameters**	Average stress score/time bin	0.19 (0.28)	0.28 (0.91)	*U *= 156.50 *p *= .242
**Affiliative behaviours**	Owner/handler proximity	0.00 (4.43)	5.41 (8.93)	*U *= 143.00 *p *= .127
	Gazing at owner/handler	7.39 (13.90)	16.64 (14.73)	*U *= 142.00 *p *= .121
	Physical contact owner	0.00 (1.99)	0.00 (1.57)	*U *= 194.00 *p *= .883
	Physical contact handler	0.00 (0.00)	0.00 (1.57)	*U *= 185.00 *p *= .698
**Other behaviours**	Barking	0.00 (0.00)	0.00 (0.00)	*U *= 198.50 *p *= .968
	Sitting	17.67 (37.06)	2.94 (31.17)	*U *= 155.50 *p *= .231
	Lying down	0.00 (0.00)	0.00 (0.00)	*U *= 179.00 *p *= .583
	Door proximity	0.00 (0.00)	0.79 (4.23)^*^	*U *= 124.00 *p *= .040
**Task-related parameters**	Number of touches needed to acquisition	10.00 (13.00)	18.00 (8.50)	*U *= 139.00 *p *= .102
	Number of no-choices	2.00 (4.50)	4.00 (4.50)	*U *= 150.00 *p *= .183
	Number of assisted trials	0.50 (1.00)	1.00 (2.00)	*U *= 162.00 *p *= .314
	Latency to acquire the behaviour (s)	102.00 (229.50)	213.50 (137.00)	*U *= 135.00 *p *= .158

* significantly different from control group at *p* < .05.

### Disruption phase

#### Stress-associated body language and overall stress levels.

The “average stress score/time bin” was significantly higher in the experimental group than in the control group. The “overall stress level”, as assessed by the observers, was also significantly higher than in the control group. See Fig 4. There was a significant positive relationship between the “overall stress level” and the “average stress score/time bin” (*r* = .51, *p* = .002).

#### Task-related behaviours.

Dogs in the control group touched the hand more frequently than dogs in the experimental group. They also tended to return to the task faster than dogs in the experimental group. See Table 6.

**Table 6:** Detailed results of the parameters measured during the Disruption phase.

		Control	Experimental	Statistics
		Median (IQR)	Median (IQR)	MWU test
**Stress-associated parameters**	Average stress score/per time bin	0.25 (0.60)	0.85 (1.02)^*^	*U *= 124.00 *p *= .040
	Overall stress level	0.00 (1.00)	1.50 (2.00)^*^	*U *= 110.00 *p *= .014
**Affiliative behaviours**	Owner proximity (%)	0.0 (2.1)	8.3 (25.0)^*^	*U = *119.00 *p = *.028
	Experimenter proximity (%)	4.2 (22.9)	0.0 (10.4)	*U *= 164.50 *p *= 0.341
	Gazing at owner/handler (%)	10.4 (14.6)	16.7 (18.8)	*U *= 156.50 *p *= .242
	Gazing at experimenter (%)	4.2 (20.8)	10.4 (22.9)	*U *= 194.00 *p *= .883
	Physical contact owner (%)	0.0 (0.0)	0.0 (4.2)	*U *= 174.00 *p *= .495
	Physical contact handler (%)	0.0 (0.0)	0.0 (0.0)	*U *= 199.50 *p *= .989
	Physical contact experimenter (%)	0.0 (2.1)	0.0 (0.0)	*U *= 177.00 *p *= .547
**Disruptor-directed behaviours**	Gazing at car (%)	33.3 (37.5)	58.3 (47.9)	*U *= 146.50 *p *= .127
	Retreating car (%)	8.3 (10.4)	8.3 (18.8)	*U *= 130.50 *p *= .060
	Approaching car (%)	2.1 (8.3)	6.3 (16.7)	*U *= 144.50 *p *= .134
	Car proximity (%)	0.00 (0.0)	0.0 (0.0)	*U *= 191.50 *p *= .820
	Door proximity (%)	0.0 (0.0)	0.0 (2.1)	*U *= 174.00 *p* = .495
**Other behaviours**	Startle (%)	0.0 (2.1)	0.0 (4.2)	*U *= 184.00 *p *= .678
	Barking (%)	0.0 (0.0)	0.0 (4.2)	*U = *183.50 *p *= .659
	Lying down (%)	0.0 (0.0)	0.0 (0.0)	*U = *187.00 *p* = .779
	Sitting (%)	4.2 (37.5)	0.0 (14.6)	*U = *137.00 *p *= .091
**Task-related behaviours**	Latency to return to the task (s)	12.00 (27.00)	23.50 (59.50)	*U* = 130.50*p* = .060
	On-task behaviours (%)	79.2 (43.8)	70.8 (70.8)	*U *= 146.50 *p *= .149
		Mean ±SD	Mean ±SD	Student’s t test
	Number of touches	12.10 (5.45)	8.40 (5.89)^*^	*t *= 2.06 *df *= 38 *p* = .046

Student’s t test was used for normally distributed data; Mann Whitney U (MWU) test was used for data that were not normally distributed.

* significantly different from control group at *p* < .05.

#### Affiliative behaviours.

Dogs in the experimental group spent a significantly higher proportion of time in the proximity of their owners than dogs in the control group. No significant differences were noted in the proportion of time bins in which other affiliative behaviours (“owner/handler proximity”, “experimenter proximity”, “gazing at owner/handler”, “gazing at experimenter”, “physical contact owner”, “physical contact experimenter”, “physical contact handler ”) were observed. See Table 6.

#### Other behaviours.

No significant differences were found in the proportion of time bins in which “barking” was observed. Similarly, there were no differences in other behaviours, like “startle”, “lying down”, “sitting”, or “door proximity”. Moreover, no differences were found in most of the behaviours linked to the disruptor (“gazing at the car”, “approaching the car”, “car proximity”). However, there was a tendency for a higher proportion of time bins where “retreating from the car” was observed in the experimental group versus the control group. See Table 6. 

### Relationships between the learning phase and the disruption phase

The relationships between “latency to acquire the behaviour” and measurements of stress in the Disruption phase (“overall stress levels”, and “average stress score/time bin”) were not significant (*ps* > .435). Similarly, no relationships were found between the “number of touches needed to acquisition” and the stress measurements in the presence of the disruptor (*ps *> .206).

The “average stress score per time bin” was higher in the Disruption phase than in the Learning phase in both groups, however, the difference was statistically significant only for the experimental group. Dogs in the experimental group also barked more and spent a higher proportion of time in the proximity of their owners in the Disruption phase than in the Learning phase. No other differences were noted in the experimental group, and in the control group, none of the coded behaviours differed significantly between the two phases (Table 7).

**Table 7:** Detailed results of the comparison of the behaviours coded in the Learning phase and the Disruption phase.

		Control			Experimental		
		Learning phase Median (IQR)	Disruption phase Median (IQR)	Wilcoxon signed-rank test	Learning phase Median (IQR)	Disruption phase Median (IQR)	Wilcoxon signed-rank test
**Stress-related parameters**	Average stress score/time bin	0.19 (0.28)	0.25 (0.60)	n = 19 *Z* = 1.83 *p* = .067	0.28 (0.91)	0.85 (1.02)^**^	n = 19 *Z* = 3.06 *p* = .002
**Affiliative behaviours**	Owner/handler proximity (%)	0.00 (4.43)	0.00 (2.08)	n = 9 *Z* = 0.77 *p* = .441	5.41 (8.93)	8.33 (25.00)^*^	n = 15 *Z* = 2.10 *p* = .036
	Gazing at owner/handler (%)	7.39 (13.90)	10.42 (14.58)	n = 19 *Z* = 1.57 *p* = .117	16.64 (14.73)	16.67 (18.75)	n = 19 *Z* = 1.61 *p* = .107
	Physical contact owner (%)	0.00 (1.99)	0.00 (0.00)	n = 7 *Z* = 1.01 *p* = .310	0.00 (1.57)	0.00 (4.17)	n = 12 *Z* = 1.10 *p* = .272
	Physical contact handler (%)	0.00 (0.00)	0.00 (0.00)	n = 8 *Z* = 0.70 *p* = .484	0.00 (0.00)	0.00 (0.00)	n = 8 *Z* = 0.56 *p* = .575
**Other behaviours**	Barking (%)	0.00 (0.00)	0.00 (0.00)	n = 5 *Z* = 0.67 *p* = .500	0.00 (0.00)	0.00 (4.17)^*^	n = 6 *Z* = 1.99 *p* = .046
	Sitting (%)	17.67 (37.06)	4.17 (37.50)	n = 15 *Z* = 0.91 *p* = .363	2.94 (31.17)	0.00 (14.58)	n = 16 *Z* = 1.86 *p* = .063
	Lying down (%)	0.00 (0.00)	0.00 (0.00)	n = 4 *Z* = 0.73 *p* = .465	0.00 (0.00)	0.00 (0.00)	n = 5 *Z* = 0.67 *p* = .500
	Door proximity (%)	0.00 (0.00)	0.00 (0.00)	n = 6 *Z* = 0.31 *p* = .753	0.79 (4.23)	0.00 (2.08)	n = 10 *Z* = 0.25 *p* = .799

* significantly different from Learning phase at *p* < .05;

** significantly different from Learning phase at *p* < .01.

## Discussion

The goal of this study was to examine the effect of a brief positive experience on learning and stress resilience in dogs. Our results do not support our hypothesis that this experience would have a positive impact. Moreover, dogs exposed to a positive experience showed higher stress levels in the presence of the disruptor than those that did not receive the positive experience. They also spent a higher proportion of time in the proximity of their owners, which can be considered as looking for reassurance [e.g., 61–63]. In a study on the effect of a positive experience on affective state in laboratory Beagles, Burman and colleagues [64] also did not observe a positive effect of a relatively similar positive experience (foraging for treats in a maze) on the outcome of a judgment bias test. Moreover, the dogs exposed to the experience showed more “pessimistic” responses than the control dogs [64]. This, in line with our findings, suggests that a brief session of positive experiences may not be sufficient to induce a sustained positive affective state in dogs and may even create a subsequent negative affective state.

There are several possible explanations for our results. Some of them highlight the possibility of our pre-treatment modulating stress susceptibility during the Disruption phase in unexpected ways. One such possibility is that experimental dogs may have been experiencing a higher level of arousal after the pre-treatment, which continued during the Learning and Disruption phases. Arousal-Valence Models [41,65] comprise a framework which categorizes emotions based on the intersection between the dimensions of arousal (from high arousal to low arousal) and valence (positive to negative). As such, if the Disruption phase was interpreted negatively (contrary to the Learning phase, where stress scores were lower and did not differ between the groups) it would be possible for highly aroused individuals to express more fearful or anxious responses in the presence of a stressor.

Another possible explanation is an unintentional induction of a negative emotional state by the termination of the positive experience when moving to the laboratory, whereas dogs in the control condition might have been experiencing boredom and/or frustration caused by lack of attention and, therefore, experienced a shift towards a positively-valenced emotional state when the “neutral” experience was terminated [64,66]. A similar result was found in sheep, which showed more optimistic approaches in a cognitive bias test after being restrained [67]. In this study, sheep from the experimental group were subjected to a 6 h restraint and isolation stress for three consecutive days and were tested daily in a judgement bias test immediately afterwards. Contrary to the initial hypothesis, sheep from the experimental group were more likely to approach the ambiguous locations compared to control sheep, who were not subjected to restraint and isolation. The authors suggested that the release from restraint immediately before testing could have resulted in a more positive emotional state. However, if this were true for the dogs in our study, we would expect to see differences in stress-related behaviours between the two groups of dogs in the first phase of the experiment (the Learning phase), but we did not see this difference.

Another alternative is that, although control dogs spent 15 min in an office space and not in the laboratory where the Learning and Disruption phases were later conducted, we cannot exclude the impact of their prolonged exposure to the smells or sounds inside of the building on their subsequent stress levels in the presence of a novel stimulus. Thus, lower stress scores in the control dogs might be associated with their longer habituation to the indoor environment, and result in a lack of heightened stress scores in this group after the introduction of the stressor. This explanation could also be supported by the fact, that dogs from the experimental group spent significantly more time in the proximity to the door (which may reflect they unwillingness to stay in the room) during the Learning phase. In an experiment comparing the behaviour of dogs and cats during a habituation test in laboratory settings (first visit to the laboratory), the authors concluded that dogs generally do not require habituation before laboratory tests, as all the participating dogs successfully met the criteria for habituation [68]. However, it is important to note that the minimum length of the habituation phase in the cited study was 5 minutes -significantly longer than the 1-minute habituation period in our study design. This discrepancy suggests that our habituation period might have been insufficient for the experimental group, especially when compared to the potential habituation experienced by the control group.

Finally, another interesting possibility could be that the waiting may have been actually stressful for control dogs, and this prior stressful experience could have prepared them to deal better with the stressor during the Disruption phase. Exposure to moderately stressful events (i.e., “stress inoculation”) has been identified as a factor promoting resilience to subsequent stressful episodes in several species [for a review, see, 69]. However, these studies usually focus on early life stressors, and more research is needed to further explore this idea. Anecdotally, it should be noted that we did not observe excessive stress signs (other than mild frustration and attention-seeking behaviours) in dogs in the control condition. However, the pre-session conditions (both the positive and the neutral experience) were not recorded, so it is not possible to formally examine the dogs’ behaviour during these events.

Another option that should be considered has to do with the delay between the pre-treatment and the Disruption phase. In the present study, experimental dogs were exposed to a positive experience before proceeding to subsequent phases of the study (Learning phase and Disruption phase). Therefore, the phase in which stress signs were analysed was preceded by a 60 s habituation phase as well as a learning task lasting 32–489 s, depending on the number of trials the dog needed to pass the acquisition criteria. As such, it is possible that this delay may have been too long for our treatment to have a positive impact on the subsequent task or that the behaviour of dogs in the Disruption phase was influenced more by the Learning phase than by the pre-treatment condition. However, this is unlikely, as we found no differences in the stress score between the groups in the Learning phase, nor a correlation between the number of trials or time needed to acquire the hand-touch behaviour and the stress scores in the Disruption phase.

Finally, we should take into account that the analysis of stress-associated behaviours in our study was based on video coding of single elements of body language (subsequently summed to a body language stress score), barking and behaviours directed toward the object, the owner, the handler and the experimenter. While the inter-observer agreement was high, there were discrepancies in the coding of body language, particularly the tail position. This is why we decided to add another measure, a holistic stress scale, which showed a moderate positive correlation with stress scores based on body language. This may have been a result of including other behaviours in the holistic assessment, like barking, retreating from the object or physical contact with the owner, as these behaviours were coded separately from the stress body language during behavioural coding. While the overall inter-rater agreement for the holistic scale was near perfect, we cannot exclude that the high agreement was a consequence of this scale being less accurate than behavioural coding. However, in some cases, dogs that achieved relatively high numbers in body language stress scores were evaluated by both observers as not stressed, which can be explained by their physical appearance (e.g., holding the tail down in a neutral position, a very short tail making the analysis of its position questionable) or panting caused by individual sensitivities to temperature or excitement associated with getting food rewards. Also, the tail position could not be assessed in the time bins in which the dog was sitting or lying down. Developing unbiased and reliable methods for evaluating behavioural responses is a big challenge in research [70,71]. Similarly, a validated observation tool for measuring affective states and welfare in dogs is still lacking [for a review, see 72]. This may point to the importance of using multiple tools (e.g., behavioural analysis and evaluation of physiological parameters) to assess dogs’ behaviour and affective states more accurately [e.g., 16,73], or using less common methods that reduce the impact of inter-rater discrepancies and bias, like crowdsourcing [74]. Additionally, qualitative methods have been implemented in recent years to facilitate the assessment of dogs’ welfare or to complete the results obtained from quantitative behavioural analyses [75,76]. Another novel approach is the use of automatic tracking and scoring of dogs’ behaviour, which reduces the risk of observer bias [77–79]. Thus, we encourage further discussion and research in this area, promoting the development of more accurate methods for evaluating animals’ emotional states.

In terms of learning effects, our results do not suggest an impact of the experimental condition on motivation and learning. It is possible that due to the characteristics of the control and experimental conditions, additional factors might have influenced the results in the subsequent tasks. These could include levels of satiety or the impact of exercise in the experimental condition. While both of these factors may be linked to perceived positive emotions, further research is needed to disentangle the possible contribution of each of these factors separately.

Limitations of our study include lack of data on the dogs’ emotional state during and after being exposed to the experimental or control condition, including the level of their arousal. Based on the previously cited literature, we chose an experience that was hoped to be positive for dogs, but the procedure itself was not recorded. Our methodology could also have resulted in erroneous findings; many assumptions had to be made such as the valence of the brief experience, the aversive quality of the toy car, and that our measures represented the concept of ‘resilience’ – all of which could be debated.

Another limitation is the lack of detailed questionnaires about some dogs who did not reach the learning criteria. For these dogs, if the owner did not fill out the questionnaire during the experiment (e.g., due to limited time), we did not collect C-BARQ scores after the experiment. While it is common to report the number of animals that were excluded from the dog cognition studies or analyses as well as the reason for exclusion [e.g., 3,28,42], possible underlying factors are rarely analysed. Unfortunately, we did not collect additional data, such as the dogs’ food motivation, that could help us analyse the factors underlying their failure.

## Conclusion

We found no positive impact of a brief positive experience on dogs’ performance in a learning task and stress resilience in the presence of a stressful stimulus. On the contrary, dogs from the control group expressed fewer stress signs in the presence of the disruptor. The most parsimonious explanation for our results is that the control group may have had a longer habituation to the indoor environment. Other explanations may be a shift toward negatively valenced emotions after terminating the positive event in dogs from the experimental group, or a “stress inoculation” of dogs in the control group. More research is needed to evaluate the effects of affective states on dogs’ behaviour during cognition tasks or in the presence of environmental challenges.

## Supporting information

S1 Dataset Raw data used for the statistical analyses. (XLSX)

## References

[1] ManyDogs Project, Espinosa J, Stevens JR, Alberghina D, Alway HEE, Barela JD, et al. ManyDogs 1: A multi-lab replication study of dogs’ pointing comprehension. Anim Behav Cogn. 2023;10(3):232–86. doi: 10.26451/abc.10.03.03.2023

[2] Hare B, Brown M, Williamson C, Tomasello M. The domestication of social cognition in dogs. Science. 2002;298(5598):1634–6. doi: 10.1126/science.1072702 12446914

[3] Miklösi Á, Polgárdi R, Topál J, Csányi V. Use of experimenter-given cues in dogs. Anim Cogn. 1998;1(2):113–21. doi: 10.1007/s100710050016 24399275

[4] Udell MAR, Dorey NR, Wynne CDL. What did domestication do to dogs? A new account of dogs’ sensitivity to human actions. Biol Rev Camb Philos Soc. 2010;85(2):327–45. doi: 10.1111/j.1469-185X.2009.00104.x 19961472

[5] Southwick SM, Bonanno GA, Masten AS, Panter-Brick C, Yehuda R. Resilience definitions, theory, and challenges: interdisciplinary perspectives. Eur J Psychotraumatol. 2014;5:10.3402/ejpt.v5.25338. doi: 10.3402/ejpt.v5.25338 25317257 PMC4185134

[6] Dziedzicka-Wasylewska M, Solich J, Korlatowicz A, Faron-Górecka A. What do the animal studies of stress resilience teach us?. Cells. 2021;10(7):1630. doi: 10.3390/cells10071630 34209787 PMC8306023

[7] Basile F, Capaccia C, Zampini D, Biagetti T, Diverio S, Guelfi G. Omics insights into animal resilience and stress factors. Animals (Basel). 2020;11(1):47. doi: 10.3390/ani11010047 33383711 PMC7824193

[8] Friedrich J, Arvelius P, Strandberg E, Polgar Z, Wiener P, Haskell MJ. The interaction between behavioural traits and demographic and management factors in German Shepherd dogs. Appl Anim Behav Sci. 2019;211:67–76. doi: 10.1016/j.applanim.2018.12.004

[9] Hakanen E, Mikkola S, Salonen M, Puurunen J, Sulkama S, Araujo C, et al. Active and social life is associated with lower non-social fearfulness in pet dogs. Sci Rep. 2020;10(1):13774. doi: 10.1038/s41598-020-70722-7 32792641 PMC7426946

[10] Ilska J, Haskell MJ, Blott SC, Sánchez-Molano E, Polgar Z, Lofgren SE, et al. Genetic characterization of dog personality traits. Genetics. 2017;206(2):1101–11. doi: 10.1534/genetics.116.192674 28396505 PMC5487251

[11] Puurunen J, Hakanen E, Salonen MK, Mikkola S, Sulkama S, Araujo C, et al. Inadequate socialisation, inactivity, and urban living environment are associated with social fearfulness in pet dogs. Sci Rep. 2020;10(1):3527. doi: 10.1038/s41598-020-60546-w 32103117 PMC7044223

[12] Bhattacharjee D, Sarkar R, Sau S, Bhadra A. Sociability of Indian free-ranging dogs (Canis lupus familiaris) varies with human movement in urban areas. J Comp Psychol. 2021;135(1):89–97. doi: 10.1037/com0000241 32584054

[13] Brubaker L, Udell MAR. The effects of past training, experience, and human behaviour on a dog’s persistence at an independent task. Appl Anim Behav Sci. 2018;204:101–7. doi: 10.1016/j.applanim.2018.04.003

[14] Paterrdale S. What is Trigger Stacking? How to Handle an Overly Stressed Dog. https://www.akc.org/expert-advice/training/how-to-avoid-trigger-stacking-in-dogs/. 2020. Accessed 2024 October 6

[15] Dogs Trust. Situation stacking. https://www.dogstrust.org.uk/how-we-help/professionals/vet-clinics/situation-stacking. Accessed 2024 October 6

[16] Beerda B, Schilder MBH, van Hooff JARAM, de Vries HW, Mol JA. Behavioural, saliva cortisol and heart rate responses to different types of stimuli in dogs. Appl Anim Behav Sci. 1998;58(3–4):365–81. doi: 10.1016/s0168-1591(97)00145-7

[17] King T, Hemsworth PH, Coleman GJ. Fear of novel and startling stimuli in domestic dogs. Appl Anim Behav Sci. 2003;82(1):45–64. doi: 10.1016/s0168-1591(03)00040-6

[18] Ley J, Coleman GJ, Holmes R, Hemsworth PH. Assessing fear of novel and startling stimuli in domestic dogs. Appl Anim Behav Sci. 2007;104(1–2):71–84. doi: 10.1016/j.applanim.2006.03.021

[19] Lensen RCMM, Moons CPH, Diederich C. Physiological stress reactivity and recovery related to behavioral traits in dogs (Canis familiaris). PLoS One. 2019;14(9):e0222581. doi: 10.1371/journal.pone.0222581 31527914 PMC6748563

[20] Protopopova A, Hall NJ, Brown KM, Andrukonis AS, Hekman JP. Behavioral predictors of subsequent respiratory illness signs in dogs admitted to an animal shelter. PLoS One. 2019;14(10):e0224252. doi: 10.1371/journal.pone.0224252 31644583 PMC6808433

[21] Duranton C, Bedossa T, Gaunet F. When facing an unfamiliar person, pet dogs present social referencing based on their owners’ direction of movement alone. Anim Behav. 2016;113:147–56.

[22] Vas J, Topál J, Gácsi M, Miklósi Á, Csányi V. A friend or an enemy? Dogs’ reaction to an unfamiliar person showing behavioural cues of threat and friendliness at different times. Appl Anim Behav Sci. 2005;94(1–2):99–115. doi: 10.1016/j.applanim.2005.02.001

[23] Stellato AC, Flint HE, Widowski TM, Serpell JA, Niel L. Assessment of fear-related behaviours displayed by companion dogs (Canis familiaris) in response to social and non-social stimuli. Appl Anim Behav Sci. 2017;188:84–90. doi: 10.1016/j.applanim.2016.12.007

[24] Bhattacharjee D, Sau S, Bhadra A. Free-ranging dogs understand human intentions and adjust their behavioral responses accordingly. Front Ecol Evol. 2018;6. doi: 10.3389/fevo.2018.00232

[25] Riggio G, Borrelli C, Campera M, Gazzano A, Mariti C. Physiological indicators of acute and chronic stress in securely and insecurely attached dogs undergoing a strange situation procedure (SSP): Preliminary results. Vet Sci. 2022;9(10):519. doi: 10.3390/vetsci9100519 36288132 PMC9610408

[26] Hiby E, Rooney N, Bradshaw J. Dog training methods: their use, effectiveness and interaction with behaviour and welfare. Anim welf. 2004;13(1):63–9. doi: 10.1017/s0962728600026683

[27] Junttila S, Valros A, Mäki K, Väätäjä H, Reunanen E, Tiira K. Breed differences in social cognition, inhibitory control, and spatial problem-solving ability in the domestic dog (Canis familiaris). Sci Rep. 2022;12(1):22529. doi: 10.1038/s41598-022-26991-5 36581704 PMC9800387

[28] Marshall-Pescini S, Valsecchi P, Petak I, Accorsi PA, Previde EP. Does training make you smarter? The effects of training on dogs’ performance (Canis familiaris) in a problem solving task. Behav Processes. 2008;78(3):449–54. doi: 10.1016/j.beproc.2008.02.022 18434043

[29] Piotti P, Szabó D, Bognár Z, Egerer A, Hulsbosch P, Carson RS, et al. Effect of age on discrimination learning, reversal learning, and cognitive bias in family dogs. Learn Behav. 2018;46(4):537–53. doi: 10.3758/s13420-018-0357-7 30251103 PMC6328011

[30] Bray EE, Otto CM, Udell MAR, Hall NJ, Johnston AM, MacLean EL. Enhancing the selection and performance of working dogs. Front Vet Sci. 2021;8:644431. doi: 10.3389/fvets.2021.644431 34055947 PMC8149746

[31] Mendl M. Performing under pressure: stress and cognitive function. Appl Anim Behav Sci. 1999;65(3):221–44. doi: 10.1016/s0168-1591(99)00088-x

[32] Cazakoff BN, Johnson KJ, Howland JG. Converging effects of acute stress on spatial and recognition memory in rodents: a review of recent behavioural and pharmacological findings. Prog Neuropsychopharmacol Biol Psychiatry. 2010;34(5):733–41. doi: 10.1016/j.pnpbp.2010.04.002 20394792

[33] McGuirk JN. State- and context-dependent learning effects in different dog populations. Master’s thesis, Virginia Polytechnic Institute and State University. 2023. https://vtechworks.lib.vt.edu/

[34] Udell MAR, Dorey NR, Wynne CDL. The performance of stray dogs (Canis familiaris) living in a shelter on human-guided object-choice tasks. Anim Behav. 2010;79(3):717–25. doi: 10.1016/j.anbehav.2009.12.027

[35] Barrera G, Fagnani J, Carballo F, Giamal Y, Bentosela M. Effects of learning on social and nonsocial behaviors during a problem-solving task in shelter and pet dogs. J Vet Behav. 2015;10(4):307–14. doi: 10.1016/j.jveb.2015.03.005

[36] Fagnani J, Barrera G, Carballo F, Bentosela M. Is previous experience important for inhibitory control? A comparison between shelter and pet dogs in A-not-B and cylinder tasks. Anim Cogn. 2016;19(6):1165–72. doi: 10.1007/s10071-016-1024-z 27541147

[37] Mendl M, Laughlin K, Hitchcock D. Pigs in space: spatial memory and its susceptibility to interference. Anim Behav. 1997;54(6):1491–508. doi: 10.1006/anbe.1997.0564 9794774

[38] Langbein J, Nürnberg G, Puppe B, Manteuffel G. Self-controlled visual discrimination learning of group-housed dwarf goats (Capra hircus): behavioral strategies and effects of relocation on learning and memory. J Comp Psychol. 2006;120(1):58–66. doi: 10.1037/0735-7036.120.1.58 16551165

[39] Doyle RE, Freire R, Cowling A, Knott SA, Lee C. Performance of sheep in a spatial maze is impeded by negative stimuli. Appl Anim Behav Sci. 2014;151:36–42. doi: 10.1016/j.applanim.2013.11.009

[40] Sümegi Z, Oláh K, Topál J. Emotional contagion in dogs as measured by change in cognitive task performance. Appl Anim Behav Sci. 2014;160:106–15. doi: 10.1016/j.applanim.2014.09.001

[41] Mendl M, Burman OHP, Paul ES. An integrative and functional framework for the study of animal emotion and mood. Proc Biol Sci. 2010;277(1696):2895–904. doi: 10.1098/rspb.2010.0303 20685706 PMC2982018

[42] Affenzeller N, Palme R, Zulch H. Playful activity post-learning improves training performance in Labrador Retriever dogs (Canis lupus familiaris). Physiol Behav. 2017;168:62–73. doi: 10.1016/j.physbeh.2016.10.014 27777045

[43] Isen AM, Daubman KA, Nowicki GP. Positive affect facilitates creative problem solving. J Pers Soc Psychol. 1987;52(6):1122–31. doi: 10.1037//0022-3514.52.6.1122 3598858

[44] Um E “Rachel,” Plass JL, Hayward EO, Homer BD. Emotional design in multimedia learning. J Educ Psychol. 2012;104(2):485–98. doi: 10.1037/a0026609

[45] Oaksford M, Morris F, Grainger B, Williams JMG. Mood, reasoning, and central executive processes. J Exp Psychol: Learn Mem Cogn. 1996;22(2):476–92. doi: 10.1037/0278-7393.22.2.476

[46] Phillips LH, Bull R, Adams E, Fraser L. Positive mood and executive function: evidence from stroop and fluency tasks. Emotion. 2002;2(1):12–22. doi: 10.1037/1528-3542.2.1.12 12899364

[47] Seibert PS, Ellis HC. A convenient self-referencing mood induction procedure. Bull Psychon Soc. 1991;29(2):121–4. doi: 10.3758/bf03335211

[48] Slyker JP, McNally RJ. Experimental induction of anxious and depressed moods: Are Velten and musical procedures necessary?. Cognit Ther Res. 1991;15.

[49] Claxton AM. The potential of the human–animal relationship as an environmental enrichment for the welfare of zoo-housed animals. Appl Anim Behav Sci. 2011;133(1–2):1–10. doi: 10.1016/j.applanim.2011.03.002

[50] Wells DL. Sensory stimulation as environmental enrichment for captive animals: A review. Appl Anim Behav Sci. 2009;118(1–2):1–11. doi: 10.1016/j.applanim.2009.01.002

[51] Murtagh K, Farnworth MJ, Brilot BO. The scent of enrichment: Exploring the effect of odour and biological salience on behaviour during enrichment of kennelled dogs. Appl Anim Behav Sci. 2020;223:104917. doi: 10.1016/j.applanim.2019.104917

[52] Mendl M, Burman OHP, Parker RMA, Paul ES. Cognitive bias as an indicator of animal emotion and welfare: Emerging evidence and underlying mechanisms. Appl Anim Behav Sci. 2009;118(3–4):161–81. doi: 10.1016/j.applanim.2009.02.023

[53] Duranton C, Horowitz A. Let me sniff! Nosework induces positive judgment bias in pet dogs. Appl Anim Behav Sci. 2019;211:61–6. doi: 10.1016/j.applanim.2018.12.009

[54] Uccheddu S, Mariti C, Sannen A, Vervaecke H, Arnout H, Gutierrez Rufo J. Behavioral and cortisol responses of shelter dogs to a cognitive bias test after olfactory enrichment with essential oils. Dog Behav. 2018;4(2):1–14. doi: 10.4454/db.v4i2.87

[55] Willen RM, Schiml PA, Hennessy MB. Enrichment centered on human interaction moderates fear-induced aggression and increases positive expectancy in fearful shelter dogs. Appl Anim Behav Sci. 2019;217:57–62. doi: 10.1016/j.applanim.2019.05.001

[56] Chan K, Arellano C, Horowitz A. Here puppy, chew on this: Short-term provision of toys does not improve welfare in companion dogs. Animals (Basel). 2023;13(21):3340. doi: 10.3390/ani13213340 37958095 PMC10648485

[57] Parker HG, Dreger DL, Rimbault M, Davis BW, Mullen AB, Carpintero-Ramirez G, et al. Genomic analyses reveal the influence of geographic origin, migration, and hybridization on modern dog breed development. Cell Rep. 2017;19(4):697–708. doi: 10.1016/j.celrep.2017.03.079 28445722 PMC5492993

[58] Hsu Y, Serpell JA. Development and validation of a questionnaire for measuring behavior and temperament traits in pet dogs. J Am Vet Med Assoc. 2003;223(9):1293–300. doi: 10.2460/javma.2003.223.1293 14621216

[59] Schipper LL, Vinke CM, Schilder MBH, Spruijt BM. The effect of feeding enrichment toys on the behaviour of kennelled dogs (Canis familiaris). Appl Anim Behav Sci. 2008;114(1–2):182–95. doi: 10.1016/j.applanim.2008.01.001

[60] Harrop A, Daniels M. Methods of time sampling: A reappraisal of momentary time sampling and partial interval recording. J Appl Behav Anal. 1986;19(1):73–7. doi: 10.1901/jaba.1986.19-73 16795692 PMC1308042

[61] Cimarelli G, Turcsán B, Bánlaki Z, Range F, Virányi Z. Dog owners’ interaction styles: Their components and associations with reactions of pet dogs to a social threat. Front Psychol. 2016;7:1979. doi: 10.3389/fpsyg.2016.01979 28066298 PMC5168437

[62] Gácsi M, Maros K, Sernkvist S, Faragó T, Miklósi A. Human analogue safe haven effect of the owner: behavioural and heart rate response to stressful social stimuli in dogs. PLoS One. 2013;8(3):e58475. doi: 10.1371/journal.pone.0058475 23469283 PMC3587610

[63] Merola I, Prato-Previde E, Marshall-Pescini S. Social referencing in dog-owner dyads?. Anim Cogn. 2012;15(2):175–85. doi: 10.1007/s10071-011-0443-0 21874515

[64] Burman O, McGowan R, Mendl M, Norling Y, Paul E, Rehn T, et al. Using judgement bias to measure positive affective state in dogs. Appl Anim Behav Sci. 2011;132(3–4):160–8. doi: 10.1016/j.applanim.2011.04.001

[65] Russell JA, Barrett LF. Core affect, prototypical emotional episodes, and other things called emotion: dissecting the elephant. J Pers Soc Psychol. 1999;76(5):805–19. doi: 10.1037//0022-3514.76.5.805 10353204

[66] Rolls ET. Emotion, motivation, decision-making, the orbitofrontal cortex, anterior cingulate cortex, and the amygdala. Brain Struct Funct. 2023;228(5):1201–57. doi: 10.1007/s00429-023-02644-9 37178232 PMC10250292

[67] Doyle RE, Fisher AD, Hinch GN, Boissy A, Lee C. Release from restraint generates a positive judgement bias in sheep. Appl Anim Behav Sci. 2010;122(1):28–34. doi: 10.1016/j.applanim.2009.11.003

[68] Uccheddu S, Miklósi Á, Gintner S, Gácsi M. Comparing pears to apples: Unlike dogs, cats need habituation before lab tests. Animals (Basel). 2022;12(21):3046. doi: 10.3390/ani12213046 36359170 PMC9657355

[69] Ashokan A, Sivasubramanian M, Mitra R. Seeding stress resilience through inoculation. Neural Plast. 2016;2016:4928081. doi: 10.1155/2016/4928081 26881112 PMC4736400

[70] Meagher RK. Observer ratings: Validity and value as a tool for animal welfare research. Appl Anim Behav Sci. 2009;119(1–2):1–14. doi: 10.1016/j.applanim.2009.02.026

[71] Tuyttens FAM, de Graaf S, Heerkens JLT, Jacobs L, Nalon E, Ott S, et al. Observer bias in animal behaviour research: can we believe what we score, if we score what we believe?. Anim Behav. 2014;90:273–80. doi: 10.1016/j.anbehav.2014.02.007

[72] de Winkel T, van der Steen S, Enders-Slegers M-J, Griffioen R, Haverbeke A, Groenewoud D, et al. Observational behaviors and emotions to assess welfare of dogs: A systematic review. J Vet Behav. 2024;72:1–17. doi: 10.1016/j.jveb.2023.12.007

[73] Palestrini C, Previde EP, Spiezio C, Verga M. Heart rate and behavioural responses of dogs in the Ainsworth’s Strange Situation: A pilot study. Appl Anim Behav Sci. 2005;94(1–2):75–88. doi: 10.1016/j.applanim.2005.02.005

[74] Root-Gutteridge H, Brown LP, Forman J, Korzeniowska AT, Simner J, Reby D. Using a new video rating tool to crowd-source analysis of behavioural reaction to stimuli. Anim Cogn. 2021;24(5):947–56. doi: 10.1007/s10071-021-01490-8 33751273 PMC8360862

[75] Arena L, Wemelsfelder F, Messori S, Ferri N, Barnard S. Development of a fixed list of terms for the qualitative behavioural assessment of shelter dogs. PLoS One. 2019;14(10):e0212652. doi: 10.1371/journal.pone.0212652 31584954 PMC6777901

[76] Walker JK, Dale AR, D’eath RB, Wemelsfelder F. Qualitative Behaviour Assessment of dogs in the shelter and home environment and relationship with quantitative behaviour assessment and physiological responses. Appl Anim Behav Sci. 2016;184:97–108. doi: 10.1016/j.applanim.2016.08.012

[77] Ren W, Wei P, Yu S, Zhang YQ. Left-right asymmetry and attractor-like dynamics of dog’s tail wagging during dog-human interactions. iScience. 2022;25(8):104747. doi: 10.1016/j.isci.2022.104747 35942104 PMC9356099

[78] Farhat N, Lazebnik T, Monteny J, Moons CPH, Wydooghe E, van der Linden D, et al. Digitally-enhanced dog behavioral testing. Sci Rep. 2023;13(1):21252. doi: 10.1038/s41598-023-48423-8 38040814 PMC10692085

[79] Völter CJ, Starić D, Huber L. Using machine learning to track dogs’ exploratory behaviour in the presence and absence of their caregiver. Anim Behav. 2023;197:97–111. doi: 10.1016/j.anbehav.2023.01.004 39045214 PMC7616271

