# Supplementary material for: Exploring the impact of a brief positive experience on dogs’ performance and stress resilience during a learning task
Source: PLoS One. 2025 Jun 23;20(6):e0326368. doi: 10.1371/journal.pone.0326368 (PMC12185004; doi:10.1371/journal.pone.0326368)
Supplement: Attachment — Submitted filename: Miller et al Review report.docx [file pone.0326368.s002.docx]

**Miller et al Review report**

This paper reports the results of an experiment conducted to test if a short positive social interaction affects the stress response caused by a sudden disruptive experience in pet dogs.

The experiment is quite elaborate and meticulously planned. The authors show that a short phase of positive experience does not enhance learning and in fact, the experimental dogs showed a higher stress response than the control dogs. They discuss several possibilities that could explain these results.

On the whole, the study is interesting, but at times, the paper is difficult to read. I suggest that the authors use a tabular format to report individual statistics, and write the results in running text. Further, the methodology would be easier to understand with the help of videos and drawings of the set-up.

While I appreciate that the authors have outlined the limitations of the study and provided several alternative explanations for their observations, this gives a feeling that they are trying to explain a result that they find unacceptable. I would recommend rewriting the discussion to some extent, keeping the possible explanations in one paragraph, but discussing the implications of the result in more detail. Since the experimental dogs showed more stress response than the control ones, can they propose an explanation for this behaviour? Are there studies on other species that could shed light on this? Could they also look closer into whether the breeds of the subjects influenced their responses? I understand that the authors are limited by the availability of subjects, but I am worried that there is too much variability among them, in terms of body size, age, food habits, in addition to the usual variability due to different life experiences.

I provide some specific comments below.

Lines 146 - 149: What were the three people in the room doing during this 15 min? Please mention this.

Line 160: Remove “that”

Figure 2 is not showing a room. This looks like the structured walk outdoors.

Please provide a drawing explaining the set-up and also a photograph.

Line 169: Were the dogs habituated to people wearing dark glasses? How can you be sure that the glasses did not affect their behaviour?

Line 292, 299, 308, 311: Please add a “0” before the decimal point all through.
