# Supplementary material for: Exploring the impact of a brief positive experience on dogs’ performance and stress resilience during a learning task
Source: PLoS One. 2025 Jun 23;20(6):e0326368. doi: 10.1371/journal.pone.0326368 (PMC12185004; doi:10.1371/journal.pone.0326368)
Supplement: Attachment — Submitted filename: Response to reviewers.docx [file pone.0326368.s004.docx]

*We would like to thank the reviewers and editor for the constructive comments we have used to refine our manuscript, including additional video coding, statistical analyses as well as extensive re-writing of the introduction and discussion section.*

*Below, we present our point-to-point answers to the reviewers’ and editor’s comments.*

**EDITOR:**

1. Please ensure that your manuscript meets PLOS ONE's style requirements, including those for file naming.

Authors: *We have made appropriate changes in the file information and headings.*

2. Thank you for stating the following financial disclosure:

"AP - Natural Sciences and Engineering Research Council of Canada Discovery Grant (RGPIN-2021-02591)"

Please state what role the funders took in the study.  If the funders had no role, please state: "The funders had no role in study design, data collection and analysis, decision to publish, or preparation of the manuscript." If this statement is not correct you must amend it as needed.

Please include this amended Role of Funder statement in your cover letter; we will change the online submission form on your behalf.

A: *We have added the information in the cover letter.*

3. Please note that funding information should not appear in the Acknowledgments section or other areas of your manuscript. We will only publish funding information present in the Funding Statement section of the online submission form. Please remove any funding-related text from the manuscript.

A: *We have removed the information on the funding from this section. We kept acknowledgements for the foundation that supported the academic exchange, as the study itself was not founded by the foundation.*

4. We note that Figures 1 and 2 includes an image of a participant in the study.

A: *The pictures are showing the setup from pilot trials, and as such are showing team members and not dog owners. We did obtain written consent to publish their images, and we now state it three times (Ethical statement, and separately for each figure (lines 120, 145, 172).*

5. Please include captions for your Supporting Information files at the end of your manuscript, and update any in-text citations to match accordingly. Please see our Supporting Information guidelines for more information.

A: *The caption is included at the end of the manuscript.*

**Reviewer #1:**

MAIN POINTS:
Exploring the effect of positive experiences in learning and stress resilience is an interesting and relevant topic, but the current manuscript suffers from a series of weaknesses at multiple levels: experimental design (e.g., no within subject baseline, unbalanced data comparisons across experimental conditions, the control condition does not seem the most appropriate), data analyses (e.g., coarse sampling of behavioural data, only a subset of these metrics are used when comparing the different experimental phases, seemingly post-hoc analysis, i.e., holistic score).

***A****uthors: Thank you for the thorough review of our study. We have made substantial changes to the manuscript, which include additional analyses (behavioural coding of videos from the learning phase as suggested, which was followed by additional data analyses – see detailed comments below. For comments on weaknesses we could not change at this stage (e.g. not having data on the dogs' emotional states during the experimental and control condition – since we have not recorded these events) we have added specific comments in the manuscript (see detailed comments below).*

Moreover, even though the authors are very thorough in detailing descriptive statistics, there is a general lack of information on all statistical tests (other than p values). Still in the statistics front, it is unclear what data were normal, and when this was not the case, why didn’t the authors try to transform it (e.g., log transform in case of latencies) before considering non-parametric alternatives.

*A: We have added the missing information on the data distribution (line 295) . The log transformation of the data (latency to acquisition, no of touches to acquisition, C-BARQ Non-social Fear subscale, C-BARQ Trainability subscale) did not result in normal data distribution; we have not decided to log transform other data because of the need for further data manipulation for data sets with “0” being a common value.*

Overall, the Introduction could use some streamlining, paragraphs read somewhat disjointedly. With regards to the Discussion, similarly to the Introduction, it lacks a clear train of thought, while it could also be more balanced in terms of discussing the data presented versus making speculative considerations that said data cannot help elucidate.

*A: We have re-written the introduction and discussion, including the removal of some points from the discussion, e.g. on the impact of different treats used in the learning task.*


DETAILED POINTS
Line 38. reference to recent ManyDogs pointing paper would be a good addition (<https://doi.org/10.26451/abc.10.03.03.2023>)

*A: we have added the citation (line 39, Reference 1)*

Lines 46, 54, 70, 75. paragraphs would be justified here

*A: The introduction was re-written taking into account all the reviews.*

Line 83. What do you mean by “Animal’s interpretation”? This is a very loaded word, consider rephrasing

Line 85. “Contains” consider “Containing”.

*A: Both these phrases were removed as we decided to remove a detailed explanation of the judgement bias test.*

Line 99. “single positive experience (including exploration, playing with a toy, and olfactory-based foraging)”
Is this really a single experience, or multiple? If the authors have reasons to support the former this should be alluded to.
Also, the stress resilience comes a bit as a surprise, maybe it would be worth adding some words beforehand, to introduce the topic to a less expert reader.

*A: We have changed “single positive experience” to “brief positive experience” throughout the manuscript, including the title.
We have also added the concept of stress-resilience in the Introduction (line 43).*

Line 111. Isn’t it simpler to say you have recruited 52 and excluded 12?

*A: We have re-written the paragraph (line 125)*

Lines 120-130. It would be nice, and easier for the reader, to have subject data summarized on a table. This would also lighten the reading of the paragraph.

*A: We have added a table summarizing the basic subject data (Table 1)*

Starting on Line 135. Consider re-structuring: after introducing the walking, segue to what the walking consisted of, and end with other details.

*A: The paragraph was re-structured as requested (line 144).*

Line 139. “did not encourage interactions”. It is unclear if this was done actively or in a passive manner. Also, it would be good to have a more detailed reasoning for the choice, duration and order of the activities used.

*A: We have added more information on the behaviour of the experimenter (line 155), as well as on the choice, duration and order of the activities (line 157)*

Lines 154-168. Consider re-structuring this paragraph. (e.g.,The setup should have been described before telling the read what it was used for.)

*A: We have re-written the paragraph (line 179), starting with the setup (leaving some information on the tasks the researchers were responsible for as it seems easier to picture this way).*

Line 162. There might be an issue with Figure 2, it does not show the task setup, it seems to be an example of the structured walk experimental animals were exposed to. Are we missing something here? Figure 1 is also never referred to in the main text.

*A: Thank you for capturing this obvious mistake. We have added a figure of the setup (Fig 1 now shows the experimental condition, Fig 2 the control condition and Fig 3 a drawing and corresponding picture of the setup)*

Line 169. We can see where the authors are coming from, but, by no means, sunglasses might prevent dogs from seeing people’s eyes, but they don’t prevent any unintentional cueing. Please either elaborate or tone down this sentence.

*A: The sentence was rephrased to: “All people were wearing dark sunglasses to prevent unintentional cueing caused by human gazing” (line 201).*

Line 173. “To increase the dogs’ motivation for this part of the task”. What do the authors mean? Isn’t the point of the study to conduct a simple reward-based learning task. The authors even say they only recruit animals that are high food motivated. Consider removing.

*A: The sentence was changed to: “The rewards were small pieces of MaplelodgeFarms® chicken hot dog” (line 204)*

Line 175. This information can be added to the Table with the subject information.

*A: We have added the information on the dogs’ IDs in the main text (line 206).*

Line 198. Can the authors be more concrete? Why was this dog excessive while all others were not, and who was this subject? If the authors what to highlight subsets of animals, then let the reader know which ones.

*A: We have added information on the dog’s ID (line 233). Since the dog was exhibiting stress constantly (barking, hiding under the chair), the session was interrupted due to welfare considerations. However, since we have counted the relative time (% of time bins, which was justified also by comparing the Learning phase with the Disruption phase, which had different durations) we did include the data of this subject in the analysis.*

Line 198. “by body language and vocalizations”. Consider adding “the” before “body language”.

*A: The word was added (line 234)*

Lines 199-203. Despite saying this phase was similar to the Learning Phase it is unclear if the experimenters kept doing trials for the duration of the Disruption.

*A: We have rewritten the paragraph so it is easier to follow and not miss the fact, that the learning task was continued during the Disruption phase (line 227)*

Line 204. This seems a side point, it is unclear what is the purpose of this. How is this a food motivation control for the disruption?

*A: We have added a statement on our reasoning for providing this additional phase: “The purpose of this phase was to ensure that the dogs' engagement (or lack of engagement) in the task was not predominantly impacted by satiety.” (line 239)*

Line 209. How were these metrics extracted, live by the helper, or from coding recorded videos.

*A: The data were extracted through the analysis of the videos.*

Line 211. As stated before, dog IDs would be valuable information for the more curious reader.

*A: We have provided the dogs’ IDs. (line 245).*

Line 213. Who is the Experimenter 1, before authors refer to experimenter and helper. Are we missing something? Please be consistent with the terminology used.

*A: We are sorry for this unintentionally inconsistent terminology. We decided to keep the terminology from the text (“the handler” and “the experimenter”) and change it in appropriate tables. As the control condition did not involve the presence of a handler (though the same team members were present in the room) we added an explanation when describing the task setup: “The owner and the handler (or one of the experimenters from the control condition, called also a handler during the task for the purpose of clarity) sat in the corner of the room.” (line 179)*

Line 216. “for this phase, videos were coded for affiliative behaviours”. For the sake of comparison, and since we believe the video data are available, why not do this for the previous learning phase as well.

*A: We have analysed the Learning phase videos and coded them for stress-related and affiliative behaviours, with the exception of gazing at the experimenter (see line 250). The results are presented in the text (line 318) and in Table 5.*

Line 218. “The coding was conducted using a partial-interval procedure with 5 s time bins. Occurrence or non-occurrence in each 5 s time bin was noted for each behaviour”. Can the authors justify this approach? Even if the authors end up binning their metrics, it is unclear why downsampling it from the start is the best way to proceed. Also, why did the authors not consider using the duration of these events instead.

*A: Although pinpoint sampling method outperforms one-zero sampling (i.e., partial interval coding), these differences are negligible given a 5 sec interval time. Nevertheless, we now recognize that, as behaviour analysts, we have “laboratory lore” that encourages the use of partial interval coding methods as tradition. We will consider using pinpoint sampling in the future.*

Line 224. As previously stated, ID of the animal is useful information.

*A: We have removed the information, as the tail position could also not be analysed for instance in time bins in which he dog was sitting. We have added a comment on that in the discussion (see line 469, 472)*

Line 226. “The lip-licking behaviour was counted as linked to stress only if it occurred in a time bin without food delivery”. Why is this the case, is it not possible that animals are stressed during food delivery? Please elaborate.

*A: During the food delivery, as the treats were moist and assumingly highly palatable, some of the dogs licked their lips immediately after receiving a piece or seemingly anticipating getting them. While stress during food delivery is possible (also when anticipating food delivery), we assumed the interpretation of this behaviour, in this case, is vague and decided to exclude the lip-licks occurring in a time bin with food delivery from the analysis.*

Line 228. “by summing up the number of stress signs in each time bin and presenting the average stress score per time bin”. Once again, the authors approach is to reduce data granularity even further without a clear justification for it. Along the same lines, what does something like “presence of stress” add to a finer measurement like “average stress score per time unit”? They are highly correlated (as can be seen from Figure 3. The latter adds no information to the former. Please consider revising the data presented in the figure as well.

*A: We have removed “Presence of stress”. In Fig 4 (previously Fig 3), we present The Overall stress (thus the result of our holistic scale, see below) and the Average stress score/time bin. Though they are correlated, the correlation is moderate and it is one of the problems we have encountered during the study, which we mention in the discussion section (line 460). We have used average stress score/time bin instead of summarizing the stress scores due to different duration of the phases (dog E16; differences in the duration of the Learning phase between dogs).*

Line 231. Table 1. Once again, the use of experimenter 1 and 2 is not consistent, early on the manuscript they were referred to as helper and experimenter. “Proximity to the car”. The definition is not clear, plus a measuring tape was never mentioned before (or after). More generally, if these descriptions were used before, a citation would be justified, if they have not, some of the description of the target behaviours should be expanded.

*A: We have carefully checked the consistency of the terminology (experimenter/handler). The ethogram was developed for this study based on the pilot trials. We have added the information on the perimeter measuring 30cm around the tub, which we missed in the text (line 187) and which is now presented also in Fig 3.*

Line 237. What exactly is the statistic used to assess Inter-observer reliability? Please elaborate.

*A: The statistics is expressed as percentage of agreement. We elaborated it in the paragraph on statistical analysis (line 305).*

Line 242. “over-interpreted as indicative of stress”. This holistic analysis can also do exactly the opposite. All definitions (Table 2) seem very post-hoc, and the lack of granularity in the measurement alone can explain the high degree of inter-observer agreement. It is unclear what this adds the analyses and interpretation of the results.

*A: We added the scale as an adjunctive measurement as it allowed us to evaluate the behaviour more holistically (as compared to coding of the body language, which for instance did not include barking or the dog’s behaviour toward the owner). We based this decision on the literature discussing different approaches to assessing behaviour in dogs ( e.g., De Winkel, T., Van der Steen, S., Enders-Slegers, M., Griffioen, R., Haverbeke, A., Groenewoud, D., & Hediger, K. (2024). Observational behaviors and emotions to assess welfare of dogs: A systematic review. Journal of Veterinary Behavior, 72, 1-17.* [*https://doi.org/10.1016/j.jveb.2023.12.007*](https://doi.org/10.1016/j.jveb.2023.12.007)*).*

*We developed our own holistic scale, as our study design was linked to very specific circumstances, that included a potential stressor the dog could interact with, the presence of the owner, handler and experimenter (which allowed the evaluation of social behaviours) as well as a task. In this case, we found that this approach is more suitable for our purposes, though we are aware that it makes the comparison with other studies, e.g using Qualitative Behavioural Assessment, impossible. We have added a comment on the scale in the discussion (line 465).*

Line 253. Unclear what the correlation was used for. Consider re-phrasing. and please add details.

*A: The manuscript was completed by adding an explanation in the paragraph discussing data analysis (line 286): “The stress level in the Disruption phase could be influenced not only by our experimental condition but also by the Learning phase, which took place directly before the Disruption phase. Therefore, a correlation was calculated to see whether there is a relationship between the “number of touches needed to acquisition” or “latency to acquire the behaviour” (which could contribute, for example, to longer habituation to the room or, on the contrary, frustration by lower success rate during the Learning phase) and the stress measurements of the Disruption phase..”*

Line 262. What were the statistics for the C-BARQ scores? t-tests? more information is needed here (e.g., t-statistics, df…). ps? How many and what tests were conducted? Were there any type of corrections for multiple comparisons? These results fall a bit short, they are not mentioned again in the manuscript.

*A: We have added more statistical data (see Table 4). We did not focus on the results, as the C-BARQ questionnaire was used only as a tool to avoid unbalanced groups, i.e. in terms of trainability of fear-based behaviours, which could influence the results of the performance in the learning task or the behaviour in the presence of a disruptor. Corrections for multiple comparisons were not used as the study involved only two groups, and in this particular case (C-BARQ) the results were non-significant already.*

Line 293. Please refer to the previous comment with regards to the stress measures used.

*A: We have added more statistical data (see Table 5,6,7).*

Line 298. “Dogs in the experimental group spent a significantly higher (U = 119.00, p = .028) proportion of time in the proximity of their owners”. As mentioned before, it would be interesting to see this data in comparison to the Learning phase.
Line 315. “Relationships between the Learning phase and the Disruption phase”. As previously stated, if this comparison is relevant, why only looking at a subset of metrics available?

*A: We have added the results obtained after coding the videos from the Learning phase (see Table 7).*

Line 323. “Our results show that this experience had no positive impact.” At least with regard to learning, absence of evidence is not evidence of absence. Please consider rephrasing.

*A: We rephrased the sentence to: “Our results do not support our hypothesis that this experience would have a positive impact.” (line 383)*

Line 331. Without a baseline metric for each dog, this is hard do assess. Moreover, in the 15 min pre-test phase, dogs are exposed to multiple things…it is unclear why this would be akin to a single rewarding event. If one uses the authors’ analyses criterium of 5 s bins, 15 minutes would be divisible into 180units, each potentially associated with rewarding events such as walking, playing, olfactory exploration.

*A: We have replaced “single” with “brief” throughout the manuscript and elaborated the information on the activities chosen when describing the experimental condition (line 157). As mentioned before (and now added as a limitation of the study, line 493) the procedure was not recorded. The cameras are set in the lab space, so both the outside area and the office space we chose for the control condition are not within the reach of the cameras. While we obviously could use portable cameras to record these events (which we will consider in the future), we have not done it for this study.*

Line 338. Consider referring to these in the Introduction as well. Yet, as it stands it is unclear how this topic can help interpreting the results, as there are no data available that would allow for it (e.g., pre-experimental baseline data).

Line 343-347. Please see general comment regarding the balance between data driven and speculative remarks.

*A: We decided to keep both these possible explanations, mentioning the Arousal-Valence Model in the discussion only, as it is only one of the possibilities we discussed. We added a comment on the fact that we have not recorded the procedure and thus could not evaluate the emotional state or the level of arousal during the two conditions (line 493).*

Line 349. Replace “can not” by “cannot”.

*A: Can not was replaced by cannot (line 419, 466)*

Line 365. “However, the pre-session conditions (both the positive and the neutral experience) were not recorded, so it is not possible to formally examine the dogs’ behaviour during these events.” This information is key to interpret the results obtained, a major flaw of the study. Please see general comment regarding experimental design.

*A: We agree that this is an unfortunate limitation of our design (as stated in line 494)*

*We based our design on previous research that did not record treatment conditions (e.g., Burman et al., 2011, where the rewarding event of foraging for treats in a maze was not recorded or evaluated, besides dogs’ unchanged motivation to take part in the task; Doyle et al., 2010, where the stressful condition was not recorded, although cortisol measures were taken as physiological parameters of stress). We realize now that having recordings of the treatments would have provided us with valuable data, and we will record such conditions in the future.*

*In terms of baseline measures, we also agree that it would have been useful to evaluate the dog’s emotional state or learning performance before the pre-session treatment. However, this was not possible because any other testing done before the session could have affected its results. One way to overcome that limitation could have been to invite participants to come to the laboratory on two different occasions. However, we decided against this possibility because it makes participation more demanding for the owners and creates some logistic difficulties, while still not providing conclusive evidence as the dog's emotional state may differ from the baseline session to the day of the test. Future studies should include designs that allow for obtaining baseline measurements as unobtrusively as possible.*

Line 409. “However, we have found a significant difference in the learning performance between dogs given commercial treats and hot dogs”. Given that the experiment was not designed to test this, this is pure speculation. Maybe this is related to other features of particular dogs, that might have caused said differences. As acknowledged by the authors, no preference tests were conducted, and there are no data regarding the animals diet, for example (Line 424).

*A: We have removed this part of the manuscript, leaving only the information on the treats we have used for dogs with allergies as we kept the number of these dogs similar in both groups.*

**Reviewer #2:**

The study investigated the impact of immediate positive experience on dogs' performance and stress resilience during a learning task. In general, the manuscript is well written with very clear methodological details and interpretation and discussion of results.

A: *Thank you for reviewing our manuscript and providing constructive comments.*

I have a few minor suggestions that can improve the clarity of the manuscript more. In particular

1) the literature cited in the current manuscript do not cover different subpopulations of dogs, so, consider exploring the work by Range and Bhadra groups on free-ranging dogs for more recent discoveries in the field of canine science.

*A: We have added some suggested references (Ref: 12, 24)*

2) Why authors did not use a randomised sequence of the learning and disruption phase?

*A: We have rewritten the paragraph so it is easier to follow and not miss the fact, that the learning task was continued during the Disruption phase (line 227). As such, we could not randomise the sequence, as first the dog had to learn the task, which was later continued during the Disruption phase.*

3) Was the amount of 'stress' (by moving a toy car) enough to elicit stress-related behaviours, which *authors also associated with 'negative emotional arousal with negative valence'.*

*A: We have added additional analyses after coding the videos from the Learning phase as well to asses the stress scores also in this phase. The results showed that the dogs were more stressed after we have started to move the car.*

4) The frequent use of 'affective states' while affect was actually not measured. Note, stress is not synonymous with affective states (like emotions and moods). I would suggest the authors to carefully review the sections where they try to explain stress with affective states.

*A: We kept the term “affective state” to describe emotional states triggered by pleasant or unpleasant experiences, trying to not use it in the context of our results from behavioural coding. While we have not measured affect, we have based the choice of the “positive experience” based on previous research.*

5) While writing two different paragraphs, the second one should be a direct continuation of the first. So, please avoid starting paragraphs with 'similarly', 'These studies', 'In addition to', etc.

*A: We have extensively rewritten both the introduction and the Discussion as suggested by the reviewers.*

6) 'A positive prior experience' sounds vague, what about replacing with 'brief'. As the positive experience period included walking on a long leash, playing with toys, etc. Practically, you cannot tease apart the effects of those events from each other (as you haven't measured affective states).

*A: We have changed “single” to “brief positive experience throughout the manuscript, including the title.*

Line 38:'human-made' sounds weird, please replace with 'human-dominated'

*A: We have applied the suggested change (line 39)*

Line 41-42: The current study did not explore genetic background associated with the behavioural responses. Therefore, I would suggest adding more relevant literature, see e.g.,

Bhattacharjee, D., Sarkar, R., Sau, S., & Bhadra, A. (2021). Sociability of Indian free-ranging dogs (Canis lupus familiaris) varies with human movement in urban areas. Journal of Comparative Psychology, 135(1), 89.

Brubaker, L., & Udell, M. A. (2018). The effects of past training, experience, and human behaviour on a dog’s persistence at an independent task. Applied Animal Behaviour Science, 204, 101-107.

*A: We have added the suggested literature (Ref: 12, 13)*

Line 45-46: see the study below that investigated how threatening may shape behaviour in dogs -

Stellato, A. C., Flint, H. E., Widowski, T. M., Serpell, J. A., & Niel, L. (2017). Assessment of fear-related behaviours displayed by companion dogs (Canis familiaris) in response to social and non-social stimuli. Applied Animal Behaviour Science, 188, 84-90.

Bhattacharjee, D., Sau, S., & Bhadra, A. (2018). Free-ranging dogs understand human intentions and adjust their behavioral responses accordingly. Frontiers in Ecology and Evolution, 6, 232.

Also see, Lazzaroni, M., Schär, J., Baxter, E., Gratalon, J., Range, F., Marshall-Pescini, S., & Dale, R. (2023). Village dogs match pet dogs in reading human facial expressions. PeerJ, 11, e15601.

*A: We have added some of the suggested literature (Ref: 23,24)*

Line 81: A 'cognitive' test..

*A: We have removed the detailed description of cognitive judgement bias test. When mentionting JBT we have added the term “cognitive” (line 102)*

Line 331-333: See my comment on pt. 6.

*A: We have changed “single” to “brief positive experience throughout the manuscript, including the title.*

**Reviewer #3**

Please see the attached file for my detailed comments.
The paper needs more clarification for better understanding of the methodology. The statistics are clear, and the language is fine. The results add to the growing understanding of dog-human interactions.

A: *Thank you for reviewing our manuscript and providing constructive comments.*

This paper reports the results of an experiment conducted to test if a short positive social

interaction affects the stress response caused by a sudden disruptive experience in pet dogs.

The experiment is quite elaborate and meticulously planned. The authors show that a short

phase of positive experience does not enhance learning and in fact, the experimental dogs

showed a higher stress response than the control dogs. They discuss several possibilities that

could explain these results.

On the whole, the study is interesting, but at times, the paper is difficult to read. I suggest that

the authors use a tabular format to report individual statistics, and write the results in running

text. Further, the methodology would be easier to understand with the help of videos and

drawings of the set-up.

*A: We have added a drawing and a picture of the setup (which we erroneously missed in the initial submission). We have also added statistical data and summarized them in the Tables, mentioning the results in the main text, as suggested.*

While I appreciate that the authors have outlined the limitations of the study and provided

several alternative explanations for their observations, this gives a feeling that they are trying

to explain a result that they find unacceptable. I would recommend rewriting the discussion to

some extent, keeping the possible explanations in one paragraph, but discussing the

implications of the result in more detail. Since the experimental dogs showed more stress

response than the control ones, can they propose an explanation for this behaviour? Are there

studies on other species that could shed light on this? Could they also look closer into

whether the breeds of the subjects influenced their responses? I understand that the authors

are limited by the availability of subjects, but I am worried that there is too much variability

among them, in terms of body size, age, food habits, in addition to the usual variability due to

different life experiences.

*A: We have rewritten the Discussion. The literature on the impact of positive experiences on learning or stress resilience in other species is rather scarce. We have added to the discussion a study on sheep, were a similarly unexpected result was found, though the animals were subjected to an unpleasant procedure prior to testing (line 413).*

*To minimize the risk of the influence of breed and the personality of dogs we have assigned the dogs to groups counterbalancing for the breed clade. We have also run the analysis of the C-BARQ subscales to make sure the groups were not significantly different in regard to trainability, attachment or fear-related behaviour, at least as reported by their owners. In terms of food habits, we kept the number of dogs reported to have food allergies/intolerance similar in both groups. Unfortunately, due to the variability of our subjects we do not have enough data to analyse the breed-related differences, as this was not a goal of this particular study.*

I provide some specific comments below.

Lines 146 - 149: What were the three people in the room doing during this 15 min? Please

mention this.

*A: We have elaborated it in the Method section (line 169)*

Line 160: Remove “that”

*A: The change was made (now line 184)*

Figure 2 is not showing a room. This looks like the structured walk outdoors. Please provide a drawing explaining the set-up and also a photograph.

*A: This was an accidental mistake, thank you for catching it. All figures including a drawing of the setup are attached in the resubmitted version.*

Line 169: Were the dogs habituated to people wearing dark glasses? How can you be sure

that the glasses did not affect their behaviour?

*A: We recruited only pet dogs. We assumed, that pet dogs are usually habituated to people wearing sunglasses on occasion, and the study was carried out during summer months/early fall. As the study design involved a learning task and a stressor we wanted to reduce the bias that might be caused by human gazing (e.g. gazing at the experimenter’s hand or gazing at the car). While it is possible, that wearing sunglasses by the owner and experimenters may impact the dogs’ behaviour, all dogs were exposed to the same procedure, thus the general impact on the data analysis across conditions is minimal.*

Line 292, 299, 308, 311: Please add a “0” before the decimal point all through.

*A: We decided to follow the APA formatting guideline and not to use “0” before the decimal point if the value (in this case, the p-value) cannot exceed 1.*
