# Supplementary material for: Exploring the impact of a brief positive experience on dogs’ performance and stress resilience during a learning task
Source: PLoS One. 2025 Jun 23;20(6):e0326368. doi: 10.1371/journal.pone.0326368 (PMC12185004; doi:10.1371/journal.pone.0326368)
Supplement: Attachment — Submitted filename: Response to reviewer.docx [file pone.0326368.s005.docx]

Reviewer #1: We thank the authors for their replies and changes to the manuscript, we believe it is greatly improved.

A: On behalf of all co-authors, I would like to thank the reviewers for dedicating their time to revise our manuscript and providing another constructive comments. Please find our detailed responses below:

Line 50. dog -> dogs

A: edited.

Line 93. consider rephrasing

A: Rephrased to: “Whereas administering pain, restraint, or isolation is easy to conceptualize as “negative” for an animal and these experiences are often included in studies on animal welfare, procedures that might induce a positive state in an animal are studied much less frequently”

Line 100. Please define/elaborate on the concept of "optimism" used in these experiments. The topic comes a bit out of nowhere, and a reader not familiar with this procedure is not provided with the appropriate information to deal with it.

A: We have removed this section from the initial version in an attempt to shorten the introduction. However, we agree that it now may be unclear to a reader, who is not familiar with JBT. We have added an elaboration (line 99)

Line 132. group -> groups

A: edited.

Figure 3. consider adding letters to refer to left-right panels.

A: We have added letters to the figure.

Line 159. Missing link to the previous statement.

A: rephrased to: “The activities were chosen, based on previous research that found that human social interaction, access to toys, and olfactory search induce ‘optimism’ in dogs [53,54]. To encourage olfactory search, and given that dogs benefit from food-stuffed toys we added treats as one of the elements of the positive experience session [59]” line 161

Line 169. When did the owners fill out the CBAR-Q in the experimental group? Before the walk?

A: We have added an explanation (line 176). Basically, we held a general conversation with the owners and also explained to them how to fill out the questionnaire. Depending on the number of questions they had, some owners managed to do it during this session, and some had to do it (or finish it) after the study. The owners of the dogs from the experimental the dogs filled out the questionnaire after the study. That is why, if the dog did not reach the learning criteria and had to be excluded from the study, we have not obtained the C-BARQ scores.

Line 176. Consider rephrasing.

A: we have edited the sentence, now line 183

Line 179. Consider rephrasing, the clarification in parentheses makes the sentence more confusing.

A: we wanted to avoid double nomenclature, that was present in the initial submission. We shortened the sentence in the parenthesis (line 186)

Line 229. This simultaneously seem hard to do, given the size of the tub and the reasoning behind it is not clear…

A: The car is small and moves easily in four directions. During the pilot studies, we have seen big differences between the sounds that can be produced by the car depending on how we operate and who is operating the car. Eventually, we have developed a unified way of moving the car back and forth and to the sides, that was possible to be kept consistent between dogs.

Line 254. Unclear what it is meant here, consider rephrasing.

A: Rephrased to: “Note that affiliative behaviours toward the experimenter were not coded in the Learning phase because it was impossible to distinguish the dog’s motivation e.g. for gazing at the experimenter at the time when they did not know the task and were engaged in learning” line 261

Line 266. This is a limitation of the study: It is not always straightforward to put a hard limit on when a behavior ends and another begins, which poses an issue when the variable is the number of such behaviors. In our view, this is the worst of both worlds: since it combines the disadvantages of binning the durations and treating behaviors like discrete units

A: We thank the reviewer for this observation. However, we believe that our text was misleading. We have now amended the text to indicate that each time bin was scored in a binary fashion (behaviour present or absent) not as counts of frequency of behaviours within that time bin. The reason we present an average stress score/time bin is the fact, that the length of the Learning phase was not consistent across dogs, and also the Disruption phase was shorter for a dog expressing excessive stress sings. This method, a partial interval coding method or one-zero sampling, has been previously used in animal behavioural studies and has the advantage of standardizing behaviours that are discreet to behaviours that are continuous. Every recording method has its advantages and disadvantages, but we believe this method allows for sufficient precision (with our length of time bins) as well as allows for comparisons across behaviours.

Table 2. the arm length -> arm’s length; closer away?

A: we have edited it in the table.

Line 272. a second observer.

A: edited

Line 275. Please elaborate.

A: We have elaborated it to: ”The highest discrepancies were seen in coding “tail down” (58.3% - 100%), therefore this behaviour was double-coded in all of the videos from the Disruption phase with a mean agreement of 96.2% ± 8.1%”. However, it is hard to say why we have seen these interpretation problems, we can only assume it is because there were some differences in interpreting the low tail position (hanging tail vs tail forcefully held down). This was one of the reasons we added the holistic scale. line 283

Line 288. there WAS a relationship.

A: edited

Line 305. This doesn't account for coincidences that may happen due to chance (as opposed to the Cohen's kappa statistic).

A: we agree. We used Cohen’s kappa for the overall stress score. For single behaviours (24 time bins for each dog, 24 different behaviours, of which some were summarized to stress score) we have decided to keep the percentages of agreement though it is for sure a less robust parameter.

Results. Given that now statistical information is presented as tables, presenting p values within the test might not be necessary. That said, if the authors prefer to do so, more information should be added regarding the tests a particular sentence refers to (e.g. t value or range of values, p or largest p)

A: we have removed the p-values for parameters, that are presented in the tables.

Line 320. No differences were significant.

A: edited

Line 324. Full stop missing before See Table 5.

A: edited

Line 326. Double full stop.

A: edited

Line 341. Remove paragraph.

A: edited

Line 378. Isn’t this a case to consider correcting for multiple comparisons? Or use a more sophisticated (e.g., GLM) model?

A: Our study set out to analyse the effects of a positive experience (pre-treatment) on subsequent learning performance (evaluated in the Learning phase) and susceptibility to stress in the presence of a disruptor (evaluated in the Disruption phase).

As such, we explored two different hypotheses.

•	Effect of pre-treatment on performance (experimental vs control in the learning phase).

•	Effect of pre-treatment on stress resilience (experimental vs control in the learning phase)

These analyses were run separately, accounting for parameters which were present on only one of the phases (e.g., behaviours related to the stressor or latency to reach the learning criterion).

We also included other parameters (like stress coding in the Learning phase) to answer some questions that arose in the review process, such as whether the remotely controlled car could be considered a stressor in the first place (for this purpose, we have used Wilcoxon rank-signed test).

We will consider a GLM approach for our next manuscript in which more groups and phases are involved.

Line 384. Double full stop.

A: edited

Line 391. Optimism and pessimism are not clearly defined (see previous comment for line 100).

A: we elaborated the definition of optimism as defined in JBT in the introduction

Line 397. This is an odd sentence in the context of a scientific paper, consider removing.

A: We removed the sentence

Line 415. More details might be helpful to make the sheep study clearer.

A: We elaborated this part of the discussion (see line 418)

Line 416. IN the Learning phase.

A: edited

Line 418. “not in the laboratory”. Is this really relevant? Or rather, is it actually true to any degree? From the moment an experiment is conducted in a room, it can be considered a laboratory, sensu lato. Maybe the same holds true for the studies cited above.

A: yes, we agree. Our point was to explain that it was not conducted in the same room/space as the learning and disruption phases. But we agree that it might not be seen as that much different by the dogs, this is why consider prolonged habituation as one of the explanations.

Line 434. What do you been by intriguing?

A: We have toned it down to: “interesting”

Line 461. But this other measure was only used for the distraction phase, which severely limits its usefulness.

A: After the first review we have attempted to recode the learning phase as well, however, we decided that this is not a useful comparison given the lack of the car, which alters the operational definition of that scale. The limitations of the holistic scale are discussed in the discussion section.

Line 473 onwards. Novel unbiased methods for automatic tracking and scoring animals’ behavior could be also mentioned in this section.

A: We have added references on this topic, see line 492, references 77-79.

Line 484. Consider rewording.

A: The sentence was shortened (line 496)

Line 496. Why is this the case?

AND
Line 500. But you did record CBAR-Q scores, what are we missing here?

A: We have explained it by: “Another limitation is the lack of detailed questionnaires from the owners of some dogs who did not reach the learning criteria. For these dogs, we did not collect C-BARQ scores after the experiment if the owner did not fill out the questionnaire during the experiment (e.g. due to limited time).” Line 510
